# Immunological characterization of a multi-epitope vaccine against *Echinococcus granulosus* based on the extracellular structural domain of CTLA-4

**DOI:** 10.3389/fimmu.2026.1947499

**Published:** 2026-10-30

**Authors:** Yanmin Li, Xizhi Ma, Haiying Jia, Jia Zheng, Nafei Chen, Wentao Zhou, Qiaoqiao Gong, Shangqi Zhao, Jianbing Ding, Xiaotao Zhou

**Affiliations:** 1 Department of Immunology, School of Basic Medical Sciences, Xinjiang Medical University, Urumqi, Xinjiang, China; 2 Clinical Laboratory Center of Xinjiang Uygur Autonomous Region People’s Hospital, Urumqi, Xinjiang, China; 3 The Fifth Affiliated Hospital of Xinjiang Medical University, Urumqi, Xinjiang, China; 4 National Infrastructures for Translational Medicine, Institute of Clinical Medicine & Peking Union Medical College Hospital, Chinese Academy of Medical Sciences and Peking Union Medical College, Beijing, China; 5 Xinjiang Key Laboratory of Molecular Biology for Endemic Diseases, Xinjiang Medical University, Urumqi, Xinjiang, China

**Keywords:** challenge experiment, CTLA-4, CTLA-4IgV-EgG1Y162-2(4), *Echinococcus granulosus*, multi-epitope vaccine

## Abstract

**Purpose:**

The aim of this study was to provide potential for the development of an efficient multi-epitope vaccine against *Echinococcus granulosus* by exploring the immunological properties and immune efficacy of the recombinant antigen CTLA-4IgV-EgG1Y162-2(4) for the effective prevention of *E. granulosus*.

**Methods:**

Target antigen sequences were retrieved from NCBI. Epitopes of CTLA-4IgV-EgG1Y162-2(4) were predicted using IEDB and SYFPEITHI, and physicochemical properties and tertiary structure were analyzed via ProtParam, ProtScale, DNASTAR, and SWISS-MODEL. The recombinant plasmid was constructed, expressed in *Escherichia coli* BL21(DE3), and purified by Ni-NTA affinity chromatography, with identity confirmed by SDS-PAGE and Western blotting. Murine dendritic cells (DCs) were cultured with the recombinant protein for 24 h and analyzed by flow cytometry for maturation and antigen binding. Balb/c mice were immunized with the recombinant protein and adjuvant; DC maturation was assessed by flow cytometry, splenocyte proliferation was evaluated by CCK-8, and serum IFN-γ, IL-4, and IgG were assessed by enzyme-linked immunosorbent assay (ELISA). Immunized mice were challenged with *E. granulosus*; after 120 days, the cyst wet weight inhibition rate was calculated, and spleen DC maturation, Th1/Th2 profiles, and serum cytokines were analyzed.

**Results:**

The amino acid sequence of CTLA-4IgV-EgG1Y162-2(4) was obtained, and epitope analysis revealed six T-cell and B-cell co-associated epitopes, with no epitope displacement caused by the CTLA-4 extracellular region or GSGGSG linker. Bioinformatics tools successfully predicted its physicochemical properties and spatial structure. The recombinant plasmid was constructed and expressed in BL21 cells. The purified protein was confirmed by His antibody and showed specific Western blot reactivity with CE-infected sera. CTLA-4IgV-EgG1Y162-2(4) promoted DC maturation both *in vitro* and *in vivo*, enhanced splenocyte proliferation, and elevated IFN-γ, IL-4, and IgG levels. In the challenge experiment, this group showed lower infection rates and cyst wet weights, and higher Th1 and lower Th2 responses, with favorable IFN-γ/IL-4 profiles.

**Conclusion:**

This study substantiates that CTLA-4IgV-EgG1Y162-2(4) stands as a multi-epitope vaccine characterized by remarkable immunogenicity and antigenicity, thus furnishing an indispensable theoretical and empirical foundation for the management and treatment of cystic echinococcosis (CE).

## 1 Introduction

Human echinococcosis is a parasitic disease caused by tapeworms of the genus *Echinococcus*. The two most important forms in humans are cystic echinococcosis (CE) and alveolar echinococcosis (AE). Echinococcosis has a worldwide epidemic distribution and is a major public health problem in South America, North Africa, Eastern Europe, the Middle East, and Central Asia (1). Human echinococcosis has been listed by the World Health Organization (WHO) as one of the 17 neglected diseases to be controlled or eliminated by 2050. CE, also known as hydatid disease or hydatidosis, is caused by infection with a species complex centered on *Echinococcus granulosus*. Humans become infected through ingestion of parasite eggs in contaminated food, water, and soil or through direct contact with animal hosts. CE can be initially screened and assisted in early diagnosis using immunological tests, where serological methods are used to detect specific antibodies against *E. granulosus* antigens, with varying degrees of sensitivity/specificity, in relation to the species involved and lesion site or the associated antigen used (2). Currently, the antigens used in immunological diagnostic reagents for the early diagnosis of echinococcosis are prone to cross-react with antibodies produced in the host against other cestodes or even trematodes, and there is a lack of better methods for early diagnosis (3). The transmission mode of *E. granulosus* is concealed and easy to spread, and the natural course of disease is long, which provides an opportunity for the pathogen to infiltrate, erode, and fully transfer. As a result, patients lack clinical symptoms in the early and middle stages of the disease, and are often at an advanced stage of the disease when symptoms appear, making the treatment process complicated. Sometimes, extensive surgery and long-term drug therapy are required. Surgical treatment combined with albendazole or mebendazole has good clinical efficacy, but there are still risks such as intraoperative transmission, postoperative recurrence, low biological utilization of drugs, side effects, and drug resistance. More critically, CE has a protracted asymptomatic latency of 5–20 years, resulting in delayed diagnosis in over 60% of symptomatic patients who already present with advanced-stage cysts (CE2/CE3b), which respond poorly to both drugs and surgery and are associated with complication-related mortality rates of 2%–5% (4). Importantly, all these interventions are downstream remedial measures that neither eliminate environmental egg contamination nor interrupt the dog–livestock–human transmission cycle. Globally, over 18,000 new CE cases occur annually, accounting for approximately 184,000 disability-adjusted life years (DALYs), with no significant decline in disease burden in highly endemic regions over the past two decades (5).

In this context, the WHO Neglected Tropical Diseases roadmap for 2021–2030 explicitly identifies echinococcosis vaccine development as a priority research area, emphasizing that an effective prophylactic vaccine represents the only fundamental tool capable of blocking oncosphere/protoscolex establishment in the liver and lungs of intermediate hosts at the source, thereby reducing primary infection rates (6). Accordingly, the vaccine strategy proposed in the present study is not intended to supplant existing therapies, but rather to serve as an upstream preventive complement—the vaccine can be inoculated into the host by artificial immunization to make the host immune system produce specific immunity against *E. granulosus*, so as to effectively cut off the transmission chain of *E. granulosus* and prevent CE (7). By protecting high-risk populations from primary exposure, it has the potential to reduce the future caseload requiring drug or surgical management and ultimately to achieve a sustained decline in CE endemicity, in addition to providing a favorable prerequisite for the plan to eradicate human cysticercosis in the 21st century.

In order to effectively prevent the spread of infectious diseases and reduce the burden on human health and socioeconomic systems, the research direction of parasitic diseases prevention and control has gradually shifted to immunoprophylaxis in recent years. The development of safe and effective vaccines to control the transmission of *E. granulosus* by cutting off the transmission cycle of *E. granulosus* pathogens through immunization of hosts such as dogs and sheep is an effective strategy for the prevention and control of *E. granulosus* (8). Compared with traditional vaccines, epitope vaccines have the advantages of high specificity, good safety, and flexible design, and can induce humoral immunity and cellular immunity at the same time, which has become a new field in the research of parasitic disease vaccines (9). Epitope refers to the specific chemical groups with various functions on the surface of antigen molecules, which can determine the specificity of antigen and efficiently induce humoral and cellular immune responses. Selecting antigen fragments rich in dominant epitopes to prepare vaccine can effectively preserve the specificity of the antigen and enhance the protective immunity of vaccine.

In the design of recombinant epitope vaccine, how to determine the epitope and linker sequence with good antigenicity is the key problem to be considered. In 2008, Cao and colleagues successfully cloned the EgG1Y162 antigen gene and sent it to GenBank, and it has been shown that the antigen is a kind of vaccine that can effectively target the terminal host of echinococcosis with strong immunogenicity (10, 11). A previous study has used biological information methods to predict the T-cell and B-cell epitope information of EgG1Y162 antigen; the results show that the EgG1Y162 recombinant antigen has a dominant distribution of linear T-cell epitopes and conformational B-cell epitopes, which can be divided into two sequences, EgG1Y162–1 and EgG1Y162-2 (12). EgG1Y162-2, which harbors more T- and B-cell epitopes, was selected for vaccine design. Linking four copies of this fragment with a GSGGSG linker was previously shown to enhance immunogenicity, and the resulting multimer EgG1Y162-2(4) elicited robust protective immunity. This study aims to develop an improved vaccine against *E. granulosus* using EgG1Y162-2(4) as the target antigen. The GSGGSG linker was chosen through multiple rounds of prediction and screening. Cytotoxic T-lymphocyte-associated protein 4 (CTLA-4/CD152), a critical immune checkpoint molecule primarily expressed on activated T cells, consists of characteristic extracellular, transmembrane, and intracellular domains. CTLA-4 shares high homology with CD28. Both receptors, located on T cells, specifically bind B7 molecules (CD80/CD86) on antigen-presenting cells (APCs). However, CTLA-4 exhibits significantly higher affinity for B7 ligands than CD28. While the CD28–B7 interaction provides a co-stimulatory signal activating T cells and promoting immune responses, CTLA-4–B7 binding delivers an inhibitory signal, suppressing T-cell activation. Although the isolated CTLA-4 extracellular domain (ECD) cannot transduce inhibitory signals across the membrane, it retains the capacity to bind APCs via its conserved B7-binding motif “MYPPPY”. The amino acid sequence “GTDDDDKAMADIGSEF” translated from the base sequence between KpnI and EcoRI on the pET30a (+) plasmid was used as a linker sequence to connect the tandem EgG1Y162-2(4) and the extracellular region of cytotoxic T lymphocyte-associated antigen 4 (CTLA-4). The 16 amino acids effectively separated CTLA-4IgV and EgG1Y162-2(4) to avoid the interference of steric hindrance, so that the recombinant protein CTLA-4IgV-EgG1Y162-2(4) could fold correctly and exert its normal immune function. Based on the above reasons, CTLA-4IgV-EgG1Y162-2(4) recombinant protein is an ideal candidate molecule for the development of *E. granulosus* vaccine. This research adopts the bioinformatics methods to predict the physicochemical properties, spatial structure. and T-cell and B-cell epitopes of CTLA-4IgV-EgG1Y162-2(4) protein, and immunological experiments were performed to verify the immune effects of this protein, which provided the necessary basis for the development of multiepitope vaccines against *E. granulosus*.

## 2 Materials and methods

### 2.1 Bioinformatics analysis

#### 2.1.1 Sequences of CTLA4-IgV-EgG1Y162-2(4) proteins

The complete amino acid sequence of EgG1Y162 was obtained from the online database of the National Center for Biotechnology Information (NCBI) (https://www.ncbi.nlm.nih.gov/). Four EgG1Y162–2 were connected using the “GSGGSG” sequence previously screened by our research group. The amino acid sequence of “GTDDDDKAMADIGSEF” on the pET30a (+) skeleton was used to link CTLA-4IgV and EgG1Y162–2 to simulate the linker sequence. Below is the plasmid map information for pET30a-CTLA-4-IgV-EgG1Y162-2(4) (**Figure 1**).

**Figure 1:**
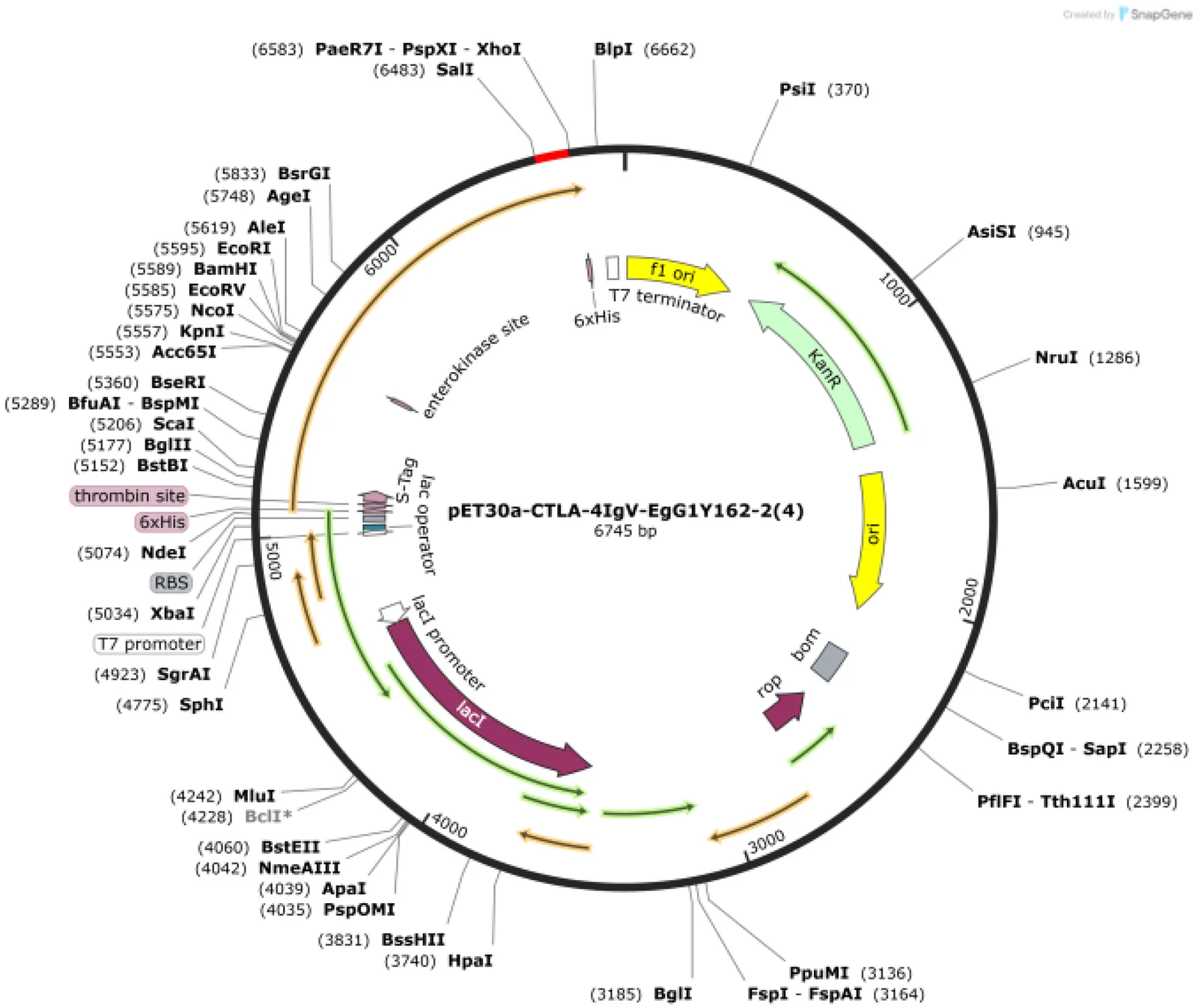
Plasmid map and sequence of pET30a-CTLA-4-IgV-EgG1Y162-2(4).

#### 2.1.2 T-cell and B-cell epitope prediction

T-cell epitopes of CTLA4-IgV-EgG1Y162-2(4) were predicted using IEDB (http://tools.iedb.org/mhcii/) and SYFPEITHI (https://syfpeithi.de/10-Home.htm) databases. Epitopes with higher scores were selected as potential dominant epitopes. B-cell epitopes can also be predicted using the online software IEDB (http://tools.iedb.org/bcell/).

#### 2.1.3 Prediction of physicochemical parameters

ProtParam online tools (https://www.expasy.org) were used to analyze CTLA4-IgV-EgG1Y162-2(4) protein molecular weight, atomic composition, theoretical isoelectric point (PI), and other physicochemical parameters. ProtScale online software (https://web.expasy.org/protscale/) was used to analyze the hydrophobicity of CTLA4-IgV-EgG1Y162-2(4) protein.

#### 2.1.4 Spatial structure prediction of recombinant proteins

The secondary structure of CTLA4-IgV-EgG1Y162-2(4) protein was predicted by DNAstar. The SWISS-MODEL online database (https://swissmodel.expasy.org/) was used for homology modeling of recombinant proteins to predict its tertiary structure. GMQE, QMEANDisCo global, and Ramachandran plot were used to evaluate the model quality.

### 2.2 Experimental assays

#### 2.2.1 Construction of recombinant plasmid pET30a-CTLA4-IgV-EgG1Y162-2(4)

The pET30a-CTLA-4IgV-EgG1Y162 and pET30a-EgG1Y162-2(4) were digested with EcoRI and HindII restriction enzymes, and the recombinant plasmid pET30a-CTLA4-IgV-EgG1Y162-2(4) was constructed by gel cutting and recovery. pET30a-CTLA-4IgV and EgG1Y162-2(4) were ligated with T4 DNA ligase at 16°C overnight, and the ligation products were transferred into competent cells DH5α. The recombinant plasmids were extracted by shaking bacteria after coating. The recombinant plasmid was digested by SalI restriction enzyme and then by BglII and HindII restriction enzymes. 1% agarose gel electrophoresis was used for preliminary verification, and the successfully verified recombinant plasmid was sent to Shanghai Sangon Biotech Co., Ltd. for sequencing.

#### 2.2.2 Expression of CTLA4-IgV-EgG1Y162-2(4) recombinant proteins in the prokaryotic system

The correctly sequenced pET30a-CTLA-4IgV-EgG1Y162-2(4) plasmid was transformed into *Escherichia coli* BL21(DE3) strain. The successfully transferred bacterial solution was inoculated in LB solid medium (containing kanamycin) and cultured inverted overnight at 37°C. The single-cell-derived colonies were cultured overnight in LB liquid medium with a kanamycin concentration of 30 μg/mL at 37°C and 220 rpm. When the bacterial solution appeared cloudy, the bacterial solution was inoculated into LB liquid medium containing kanamycin at a ratio of 1:50. When *A*_600_ reached 0.6 to 0.8, 0.7 mmol/L IPTG (isopropyl β-D-thiogalactoside) (Solarbio Technology Co., Ltd., Beijing, China) was added and cultured at 37°C for 4 h to induce the expression of recombinant protein. After ultrasonic crushing, the supernatant was obtained by 12,000 rpm and centrifugation for 10 min. The protein was purified by nickel column affinity chromatography, and the recombinant protein was competitively eluted by imidazole buffer after the column. To remove endotoxin and non-specifically bound contaminating proteins, the Ni-NTA resin was washed with an imidazole washing buffer containing 60% isopropanol, so as to obtain the highly purified His-tag protein. The expression and purification of CTLA-4IgV-EgG1Y162-2(4) protein in bacterial supernatant were analyzed by SDS-PAGE. His-tag protein CTLA-4IgV-EgG1Y162-2(4) was identified by Western blot. The primary antibody was murine-derived His-tag monoclonal antibody (1:1,000 dilution) and incubated at 4°C overnight. For the secondary antibody, horseradish peroxidase (HRP)-labeled sheep anti-mouse antibody (1:2,000 dilution) was used and incubated at room temperature in the dark for 2 h. After washing the membrane with TBST, ECL was added and the results were observed and recorded.

#### 2.2.3 Specific antibody was detected by western blot

Western blot assay was used to detect the specific antibody response against CTLA-4IgV-EgG1Y162-2(4) recombinant protein in sera of patients infected with *E. granulosus* and mice infected with *E. granulosus*. Uninfected people and normal mice served as controls. The recombinant proteins were transferred to PVDF membranes (Merck & Co., America) with a pore size of 0.45 μm by 12% SDS-PAGE gel electrophoresis. The membrane was enclosed in 5% skim milk powder solution for 2 h at room temperature and washed three times with Tris-hydrochloric acid buffer (TBST) for 15 min each time. PVDF membranes were incubated overnight in a 4°C shaker using normal sera, sera of patients with *E. granulosus* (1:400 dilution), sera of normal mice, and sera of mice infected with *E. granulosus* (1:300 dilution). Then, they were washed three times with TBST and subsequently treated with HRP conjugated with goat anti-mouse IgG with 1:2,000 dilution and goat anti-human IgG-HRP (Absin Biotechnology Co., Shanghai, China) with 1:4000 dilution and incubated at room temperature in the dark for 2 h. The protein bands were observed after washing the PVDF membrane three times with TBST.

#### 2.2.4 Co-culture of mouse bone marrow-derived DCs with recombinant proteins

Female C57 mice (6–8 weeks old) were purchased from the Experimental Animal Center of Xinjiang Medical University. The mouse bone marrow cells were isolated after sacrifice by cervical dislocation. Cells were suspended in RPMI-1640 complete culture medium containing penicillin/streptomycin, rmGM-CSF at a final concentration of 10 ng/mL, and rmIL-4 at 20 ng/mL; gently mixed; and counted. The cells were diluted into a 3 × 10^6^ cell suspension using RPMI-1640 complete culture medium and inoculated into six-well plates, which were divided into a control group, an EgG1Y162-2(4) group, and a CTLA4-IgV-EgG1Y162-2(4) group, and placed in a 37°C and 5% CO_2_ incubator for culture. The morphological changes of cells were observed on the second, fourth, and seventh days. On the sixth day of stimulus induction, 500 ng/mL of EgG1Y162-2(4) protein was added to each well in the EgG1Y162-2(4) group, and the same concentration of CTLA4-IgV-EgG1Y162-2(4) protein was added to each well in the CTLA4-IgV-EgG1Y162-2(4) group. After 24 h, the binding rate and maturity of dendritic cell (DC) in the EgG1Y162-2(4) group and the CTLA4-IgV-EgG1Y162-2(4) group were detected. The cells of each group were collected and centrifuged at 1,000 rpm, and the supernatant was discarded. PBS (1.5 mL) was added to each tube, gently blown and mixed, and centrifuged again at 1,000 rpm; the supernatant was discarded. In the DC maturity assay group, 50 μL of PBS containing 1 μL of CD45 antibody, 1 μL of CD11c antibody, and 1 μL of I-Ab antibody (BD Biosciences, Franklin Lakes, USA) was added. In the protein-binding group, 50 μL of PBS containing 1 μL of FITC-HiS antibody (BD Biosciences, Franklin Lakes, USA) was added. After incubation at 4°C for 30 min, each tube was washed with 400 μL of MACS and centrifuged at 1,000 rpm for 5 min, and the supernatant was discarded. MACS (300 μL) was added to the suspended cells, and cytokine expression was detected within 4 h. At the same time, a blank tube and a single dye tube were set.

#### 2.2.5 Vaccine immunization in BALB/c mice

Twenty-four SPF grade female BALB/c mice aged 6 weeks, weighing 20 ± 2 g, were reared at 23 ± 3°C and at a relative humidity of 40%–70%. The mice were divided into four groups with six mice in each group. The mice in the normal control group were fed normally and did not receive any injection. Mice in the negative control group received subcutaneous injections of the same volume of saline at multiple points in the back. An adjuvant-only control group was not included in this study. Freund’s complete adjuvant (FCA) alone causes persistent ulceration and pain in mice, which raises significant animal welfare concerns. In addition, space constraints in the animal facility limited the number of experimental groups. Therefore, this control group was omitted in accordance with institutional animal ethics guidelines. The mice in the EgG1Y162-2(4) immunized group were injected subcutaneously at multiple points on the back, and 300 μL of the suspension of EgG1Y162-2(4) protein and FCA was injected for the first immunization, and the suspension of EgG1Y162-2(4) protein and Freund’s incomplete adjuvant was given for the second and third immunization 14 and 28 days later. The mice in the CTLA-4IgV-EgG1Y162-2(4) immunized group were injected subcutaneously at multiple points on the back, and 300 μL of CTLA-4IgV-EgG1Y162-2(4) protein and FCA suspension was injected for the first immunization. CTLA-4IgV-EgG1Y162-2(4) protein and Freund’s incomplete adjuvant suspension were immunized for the second and third times on days 14 and 28. Approximately 100 μL of orbital blood was collected once a week in each group during immunization, and serum was separated and stored. After 43 days, mice were sacrificed and spleen tissues were collected to prepare suspension of mouse spleen cells (**Figure 2**).

**Figure 2:**
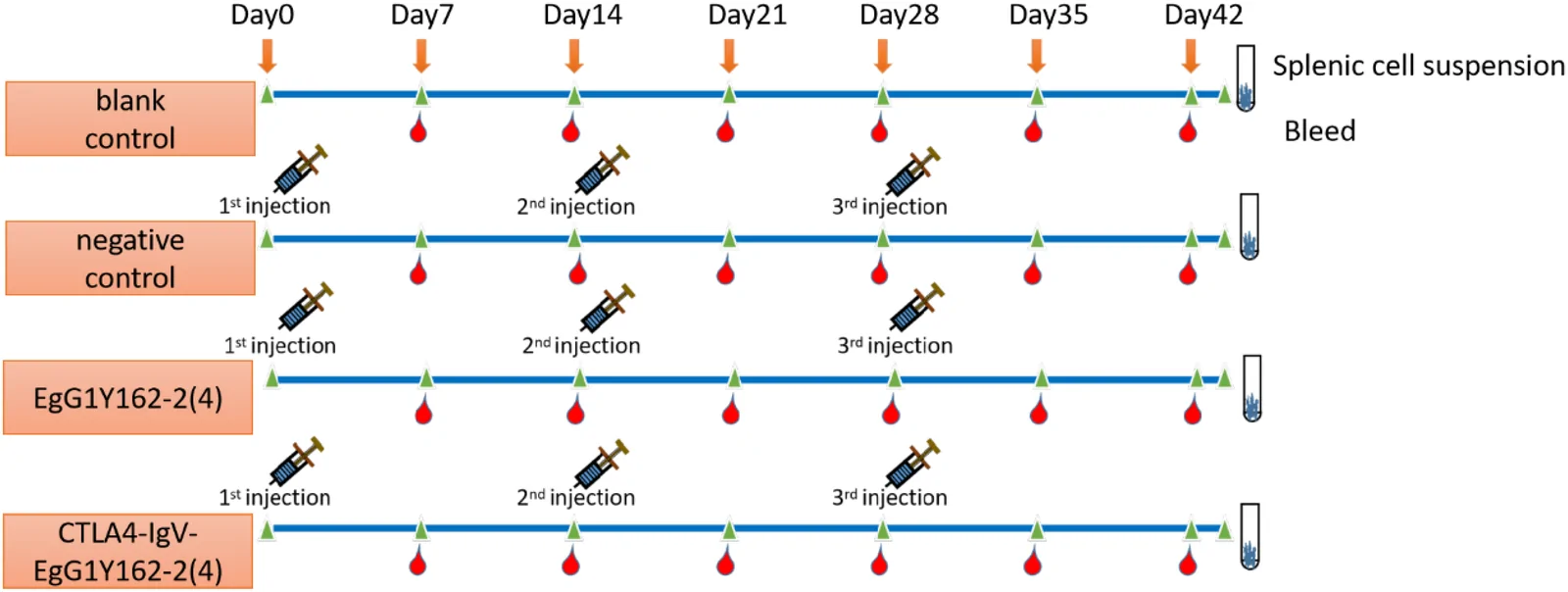
Schematic diagram of subcutaneous immunization and sample collection schedule. Balb/c mice were immunized subcutaneously on days 0, 14, and 28, and blood samples were collected weekly. Spleen tissues of all mice were collected on day 43.

##### 2.2.5.1 Assessment of dendritic cell maturation in murine spleen utilizing flow cytometry

Spleen cell suspension of mice in each group was prepared with PBS, and the cell concentration was adjusted to 1 × 10^6^/mL. CD11c antibody (1 μL), 0.5 μL of I-Ab antibody, and 0.625 μL of CD86 antibody were added successively, incubated at 4°C for 20 min in the dark, and then detected by a flow cytometer. The maturation of DC cells in spleen tissues of mice in each group was observed and analyzed.

##### 2.2.5.2 Evaluation of mouse spleen cell proliferation using the CCK-8 assay

Spleen cell suspension of mice in each group was prepared with RPMI-1640 medium containing 10% BSA, and cell concentration was adjusted to 5 × 10^6^/mL. The cells were added to 96-well plates and cultured for 48 h. The normal control group was not stimulated, while the negative control group was stimulated with PBS. EgG1Y162-2(4) and CTLA-4IgV-EgG1Y162-2(4) groups were supplemented with 5 μg/mL recombinant protein EgG1Y162-2(4) and CTLA-4IgV-EgG1Y162-2(4), respectively. After 48 h of stimulation, CCK-8 was used to detect the proliferation of spleen cells in each group.

##### 2.2.5.3 Evaluation of cytokines by ELISA

Splenic cell suspension of mice in each group was prepared with the medium containing 10% BSA, the cell concentration was adjusted to 5 × 10^6^/mL, and cells were cultured in 96-well plates for 48 h. The normal group was not stimulated, while the negative control group was stimulated with PBS. The EgG1Y162-2(4) group and the CTLA-4IgV-EgG1Y162-2(4) group were readded with 5 μg/mL recombinant protein EgG1Y162-2(4) and CTLA-4IgV-EgG1Y162-2(4), respectively. After 48 h of stimulation, cell supernatant of each group was collected. The contents of interleukin-4 (IL-4) and interferon-γ (IFN-γ) in the supernatant of spleen cells of mice in each group were determined by enzyme-linked immunosorbent assay (ELISA) according to the kit instructions (MultiSciences, Hangzhou, China).

##### 2.2.5.4 Determination of specific antibody titers via ELISA

Serum-specific protective antibody IgG secretion was detected by ELISA. EgG1Y162-2(4) protein was diluted to 5 μg/mL with coating solution, coated in an ELISA plate with 0.1 mL/well, and placed in a refrigerator at 4°C overnight. The serum of immunized mice in each group and goat anti-mouse IgG labeled by HRP were used as antibodies. The absorbance values at 450 nm were read to detect the antibody titers of mice in each group.

#### 2.2.6 Immunization of BALB/c mice with recombinant proteins EgG1Y162 and CTLA-4-IgV-EgG1Y162-2(4) followed by challenge infection with *Echinococcus granulosus*

Following three immunizations with the vaccine, BALB/c mice were intraperitoneally challenged with protoscoleces of *E. granulosus* to establish an artificial infection, thereby evaluating the preventive/protective efficacy of the vaccine in mice. Protoscoleces of *E. granulosus* used in this study were isolated from the livers of sheep infected with *E. granulosus*, which were obtained from a local sheep and cattle slaughterhouse in Midong District, Urumqi, Xinjiang Uygur Autonomous Region, China. The slaughtering procedures were conducted under the supervision and inspection of authorized personnel throughout the process, in accordance with local animal slaughter regulations.

##### 2.2.6.1 Immunization of BALB/c mice with recombinant proteins

Twenty-four specific pathogen-free (SPF) female BALB/c mice, aged 6–8 weeks and weighing 20 ± 2 g, were randomly divided into four groups: a control group (*n* = 6), an Eg model group (*n* = 6), an EgG1Y162 group (*n* = 6), and a CTLA-4-IgV-EgG1Y162-2(4) group (*n* = 6). The mice were identified by ear tagging. The control group received no treatment. For the immunized groups, the primary immunization was administered by subcutaneous injection of the protein vaccine emulsified in FCA. At the third and fifth weeks after the primary immunization, booster immunizations were given subcutaneously using the protein vaccine emulsified in Freund’s incomplete adjuvant. Two weeks after the third immunization, the Eg model group and the immunized experimental groups were challenged intraperitoneally with protoscoleces of *E. granulosus* to establish infection.

##### 2.2.6.2 Preparation of *Echinococcus granulosus* protoscolex suspension

Protoscoleces were obtained from the cyst fluid of fresh sheep livers infected with *E. granulosus* by precipitation on ice. The cyst wall was dissected, minced, and sequentially filtered through 40-mesh and 100-mesh sieves, and the filtrate was also precipitated on ice. The protoscolex pellets from both the cyst fluid and cyst wall were combined. One milliliter of the pellet was mixed with 10 mL of pepsin solution (containing 0.5 g of pepsin and 200 μL of concentrated hydrochloric acid, adjusted to 50 mL with ddH_2_O, and filtered through a 0.22-μm filter), then incubated in a 37 °C water bath for 30 min with shaking every 5 min. After digestion, the supernatant was discarded, and the protoscoleces were washed three times with physiological saline, filtered through a 100-μm mesh, and washed twice with physiological saline containing 2% penicillin–streptomycin. Protoscoleces were counted under a microscope, diluted to a concentration of 2.5 × 10^4^ protoscoleces/mL, aliquoted into 1.5-mL Eppendorf tubes (200 μL/tube), and kept on ice for subsequent intraperitoneal injection into mice.

##### 2.2.6.3 Intraperitoneal infection of mice with *Echinococcus granulosus* protoscoleces

The pre-aliquoted protoscolex suspension was intraperitoneally injected into mice of the Eg model group and experimental groups at a volume of 200 μL per mouse (containing 5,000 protoscoleces). At 120 days post-infection, some mice in the Eg model group were euthanized to assess the infection status of the mouse model. When the infection rate reached 80%, all mice were weighed, euthanized by cervical dislocation, and subjected to laparotomy for observation and gross photography of abdominal and hepatic infections. The livers and hydatid cysts were collected, weighed, and photographed for documentation.

##### 2.2.6.4 Detection of Th1 and Th2 cell percentages in the spleens of mice following challenge infection by flow cytometry

Spleens were collected from mice, washed in a Petri dish containing FACS buffer, and ground through a 200-mesh stainless steel sieve. After centrifugation, red blood cells were lysed with 1× RBC lysis buffer, and the reaction was terminated with FACS buffer. Cells were resuspended in complete RPMI-1640 medium supplemented with 10% FBS, counted, and adjusted to a concentration of 2 × 10^6^ cells/mL (200 μL). Leukocyte Activation Cocktail was added, and cells were stimulated for 4–6 h. Following stimulation, cells were centrifuged, washed, and incubated with FACS buffer containing FITC-CD3 and APC-CD4 at 4 °C in the dark for 30 min. After washing, cells were fixed with fixation/permeabilization buffer at 4 °C for 1–2 h. After two washes with 1× wash buffer, cells were incubated with 1× wash buffer containing PE-IFN-γ and PE-Cy7-IL-4 at room temperature in the dark for 1 h. Following a final wash, the cell suspension was filtered through a 200-mesh sieve into flow cytometry tubes and analyzed. Blank and single-stained controls were prepared accordingly.

##### 2.2.6.5 ELISA detection of IL-4 and IFN-γ secretion levels in serum of challenge-infected mice

The detection method was identical to that in section 2.2.5.3.

### 2.3 Statistical analysis

GraphPad Prism 8.3.0 software was used for statistical analysis and plot. Data were expressed as mean ± standard deviation (SD), one-way analysis of variance (ANOVA) was used for comparison between groups, and independent sample *t*-test was used for comparison between two independent groups. *p-*value less than 0.05 was considered as significant difference. *p < 0.05, **p < 0.01, ***p < 0.001, and ****p < 0.0001.

## 3 Results

### 3.1 Results of bioinformatics analysis

#### 3.1.1 The amino acid sequences of proteins

The accession of EgG1Y162 protein was BAH30229 and the version was BAH30229.1. The amino acid sequence was VDPELMAKLTKELKTTLPEHFRWIHVGSRSLELGWNATGLANLHADHIKLTANLYTTYVTFKYRNVPIERQKLTLEGLKPSTFYEVVVQAFKGGSQVFKYTGFIRTLAPGEDGADRASGF. EgG1Y162–2 is the 52–120 amino acid of EgG1Y162 protein, with 69 amino acids in its total length. The amino acid sequence was ANLYTTYVTFKYRNVPIERQKLTLEGLKPSTFYEVVVQAFKGGSQVFKYTGFIRTLAPGEDGADRASGF. The amino acid sequence of CTLA-4IgV-EgG1Y162-2(4) was NVTQPPVVLASSRGVASFTCEYESSGKADEVRVTVLRKAGIQVTEVCAGTYMVEDELTFLDDSSCIGTSRGNKVNLTIQGLRAMDTGLYVCKVELMYPPPYYMGEGNGTQIYVIDPEPCPDSDGTDDDDKAMADIGSEFANLYTTYVTFKYRNVPIERQKLTLEGLKPSTFYEVVVQAFKGGSQVFKYTGFIRTLAPGEDGADRASGFGSGGSGANLYTTYVTFKYRNVPIERQKLTLEGLKPSTFYEVVVQAFKGGSQVFKYTGFIRTLAPGEDGADRASGFGSGGSGANLYTTYVTFKYRNVPIERQKLTLEGLKPSTFYEVVVQAFKGGSQVFKYTGFIRTLAPGEDGADRASGFGSGGSGANLYTTYVTFKYRNVPIERQKLTLEGLKPSTFYEVVVQAFKGGSQVFKYTGFIRTLAPGEDGADRASGFVD.

#### 3.1.2 Prediction of dominant T-cell and B-cell epitopes for CTLA4-IgV-EgG1Y162-2(4)

T-cell epitope prediction is based on MHC peptide binding, whereas B-cell epitope prediction requires consideration of the sequence and 3D structure of the antigen (13–15). T-cell epitope and B-cell epitope of CTLA4-IgV-EgG1Y162-2(4) were predicted by IEDB and SYFPEITHI online software. The two prediction software have different scoring systems, but both can predict potential epitopes according to the score. The higher the prediction score, the more likely it is a potential T-cell epitope or B-cell epitope. In order to achieve better antigenicity of epitopes, three overlapping epitope regions of T cells and B cells were screened out according to the predicted results, and the B-cell antigen epitopes of CTLA4-IgV-EgG1Y162-2(4) were analyzed by IEDB software. The epitopes with high predicted scores that overlapped with B-cell epitopes were 52–62, 117–134, 197–215, 272–290, 347–365, and 422–432, and the epitopes of relative dominance of T cells were 48–62, 119–133, 193–207, 268–282, 343–357, and 418–432. The T-cell epitope of CTLA4-IgV-EgG1Y162-2(4) was predicted to be 48–62, 129–143, 188–202, 263–277, 338–352, and 413–427 by the SYFPEITHI online software (**Table 1**). Combined with the prediction results of the two databases, we screened out the potential dominant epitopes of T cells and B cells as 52–62, 119–133, 197–207, 272–282, 347–357, and 422–432 (**Table 2**). It is essential to highlight that the T-cell and B-cell association epitopes of CTLA4-IGV-EgG1Y162-2(4) remained unaltered following its linkage to EgG1Y162-2, facilitated by the “GSGGSG” linker sequence.

**Table 1:** Predictions of T-cell and B-cell epitopes by IEDB and SYFPEITHI.

Web servers	Epitopes	Position	Amino acid sequences	Score
IEDB	TB-cell epitopes	52–62 117–134 197–215 272–290 347–365 422–432	MVEDELTFLDD EPCPDSDGTDDDDKAMAD PGEDGADRASGFGSGGSGA PGEDGADRASGFGSGGSGA PGEDGADRASGFGSGGSGA PGEDGADRASG	11 18 19 19 19 11
	T-cell epitopes	48–62 119–133 193–207 268–282 343–357 418–432	AGTYMVEDELTFLDD CPDSDGTDDDDKAMA RTLAPGEDGADRASG RTLAPGEDGADRASG RTLAPGEDGADRASG RTLAPGEDGADRASG	80 99 99 99 99 99
SYFPEITHI	T-cell epitopes	48–62 129–143 188–202 263–277 338–352 413–427	AGTYMVEDELTFLDD DKAMADIGSEFANLY YTGFIRTLAPGEDGA YTGFIRTLAPGEDGA YTGFIRTLAPGEDGA YTGFIRTLAPGEDGA	22 26 18 18 18 18

**Table 2:** Predictions of T-cell and B-cell combined epitopes.

Position		Sequence
B-cell antigen epitope	T-cell antigen epitope	T-cell and B-cell combined epitopes
52–62 117–134 197–215 272–290 347–365 422–432	48–62 119–133 193–207 268–282 343–357 418–432	AGTY***MVEDELTFLDD*** EP***CPDSDGTDDDDKAMA***D RTLA***PGEDGADRASG***FGSGGSGA RTLA***PGEDGADRASG***FGSGGSGA RTLA***PGEDGADRASG***FGSGGSGA RTLA***PGEDGADRASG***

Bold indicates B-cell antigen epitopes.

#### 3.1.3 Physicochemical properties of CTLA4-IgV-EgG1Y162-2(4) recombinant protein

ProtParam online database shows that CTLA-4IgV-EgG1Y162-2(4) protein was composed of 435 amino acids, and protein relative molecular weight was 47,318.22. Theoretical PI was 5.67, the chemical formula was C2125H3267N551O654S10, and the extinction coefficient was 40,480. The instability index (II) was computed to be 24.54 (when the value <40, the protein is stable) and the average hydrophilic coefficient is GRAVY at −0.264 (the value of GRAVY ranges from −2 to 2). The results of the ProtScale online database showed that the 126th amino acid had the lowest score (−2.589), and the 175th, 250th, 325th, and 400th amino acids had the highest score (1.300); a higher score indicates a stronger hydrophobicity, and the hydrophobicity results are shown in **Figure 3A**.

**Figure 3:**
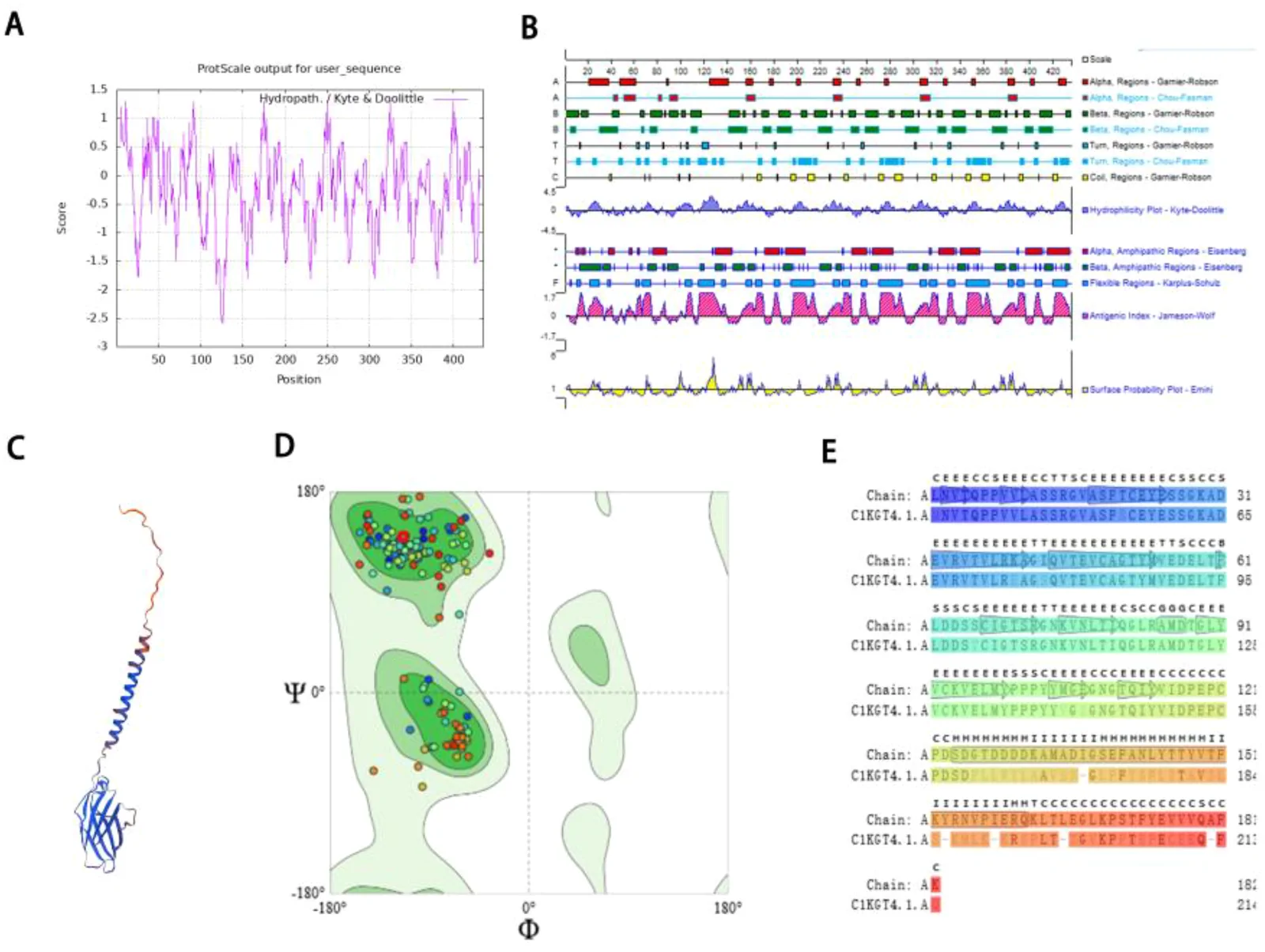
Biological properties of CTLA4-IgV-EgG1Y162-2(4) protein predicted by bioinformatics software. **(A)** Hydrophobicity of CTLA4-IgV-EgG1Y162-2(4) predicted by ProtScale. **(B)** The secondary structure of CTLA-4IgV-EgG1Y162-2(4) protein predicted by DNAstar-Protean. **(C)** Tertiary structure of CTLA4-IgV-EgG1Y162-2(4) protein predicted by SWISS-MODEL. **(D)** Ramachandran plot. **(E)** Alignment.

#### 3.1.4 Spatial structure of CTLA4-IgV-EgG1Y162-2(4) recombinant protein

The structure diagram of CTLA4-IgV-EgG1Y162-2(4) protein was obtained by the Garnier–Robson and Chou–Fasman methods in DNAstar software (**Figure 3B**). The homology model of CTLA4-IgV-EgG1Y162-2(4) was constructed by the SWISS-MODEL online software, exhibits no structural deviation, and maintains proper folding (**Figure 3C**), and the sequence identity is 75%. GMQE gives an overall model quality measurement between 0 and 1, with higher numbers indicating higher expected quality. The model structure prediction results of CTLA4-IgV-EgG1Y162-2(4) recombinant protein showed that the GMQE value was 0.34. The model quality was assessed as shown in **Figure 3D**. The results of sequence alignment are shown in **Figure 3E**. The results of quality evaluation showed that the constructed spatial structure model of CTLA4-IgV-EgG1Y162-2(4) recombinant protein was reliable.

### 3.2 Results of the experiment

#### 3.2.1 Identification of pET30a-CTLA4-IgV-EgG1Y162-2(4) plasmid by enzyme digestion

After the constructed plasmid was digested by single enzyme and double enzyme, the results of agarose gel showed that a linear band appeared in the single enzyme product, and the molecular weight was in line with the expected results (**Figure 4A**). After double digestion, the plasmid skeleton was cut into two bands, the molecular weight of which was approximately 5,354 and 1,312 bp, respectively (**Figure 4B**). The size of the digested fragment was in line with the expected results.

**Figure 4:**
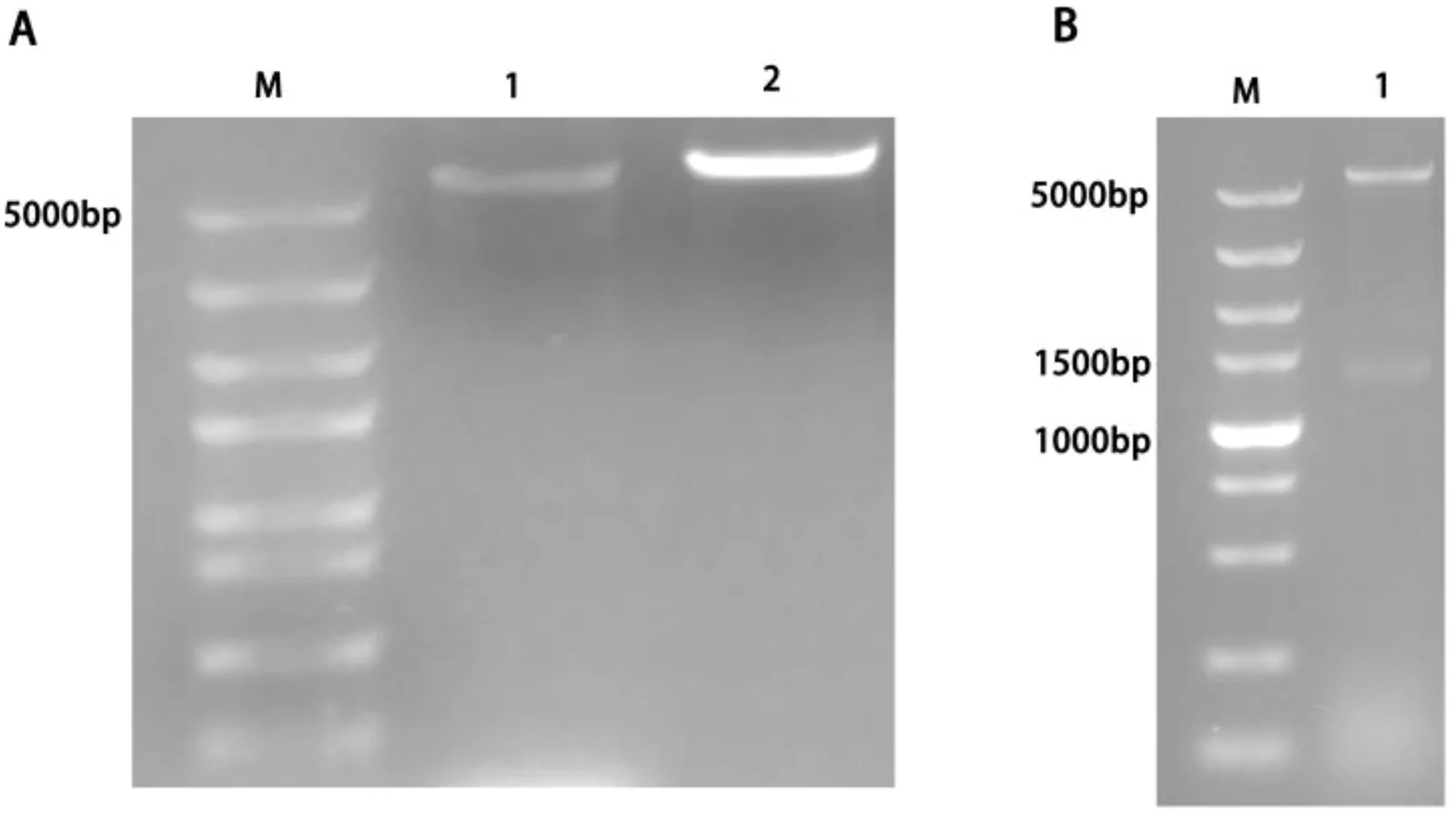
Enzymatic identification of pET30a-CTLA4-IgV-EgG1Y162-2(4) recombinant plasmid. M: DNA marker. **(A)** Single enzyme digestion identification. Lane 1: Control plasmid; Lane 2: SalI restriction endonuclease enzyme cuts plasmid. **(B)** Double enzyme digestion identification. Lane 1: The recombinant plasmid was digested by BglII and HindII restriction enzymes.

#### 3.2.2 Prokaryotic expression and purification of CTLA4-IgV-EgG1Y162-2(4) protein

SDS-PAGE analysis of the induction and purification of CTLA4-IgV-EgG1Y162-2(4) protein showed that the target band appeared at a molecular weight of 53 kDa, indicating that one CTLA4-IgV-EgG1Y162-2(4) protein was successfully expressed in *E. coli* BL21(DE3), and the purified CTLA4-IgV-EgG1Y162-2(4) protein was obtained by nickel column affinity chromatography. The target band was successfully detected with HIS antibody at 53-kDa molecular weight, indicating that the purified CTLA4-IgV-EgG1Y162-2(4) protein was successfully obtained (**Figure 5**).

**Figure 5:**
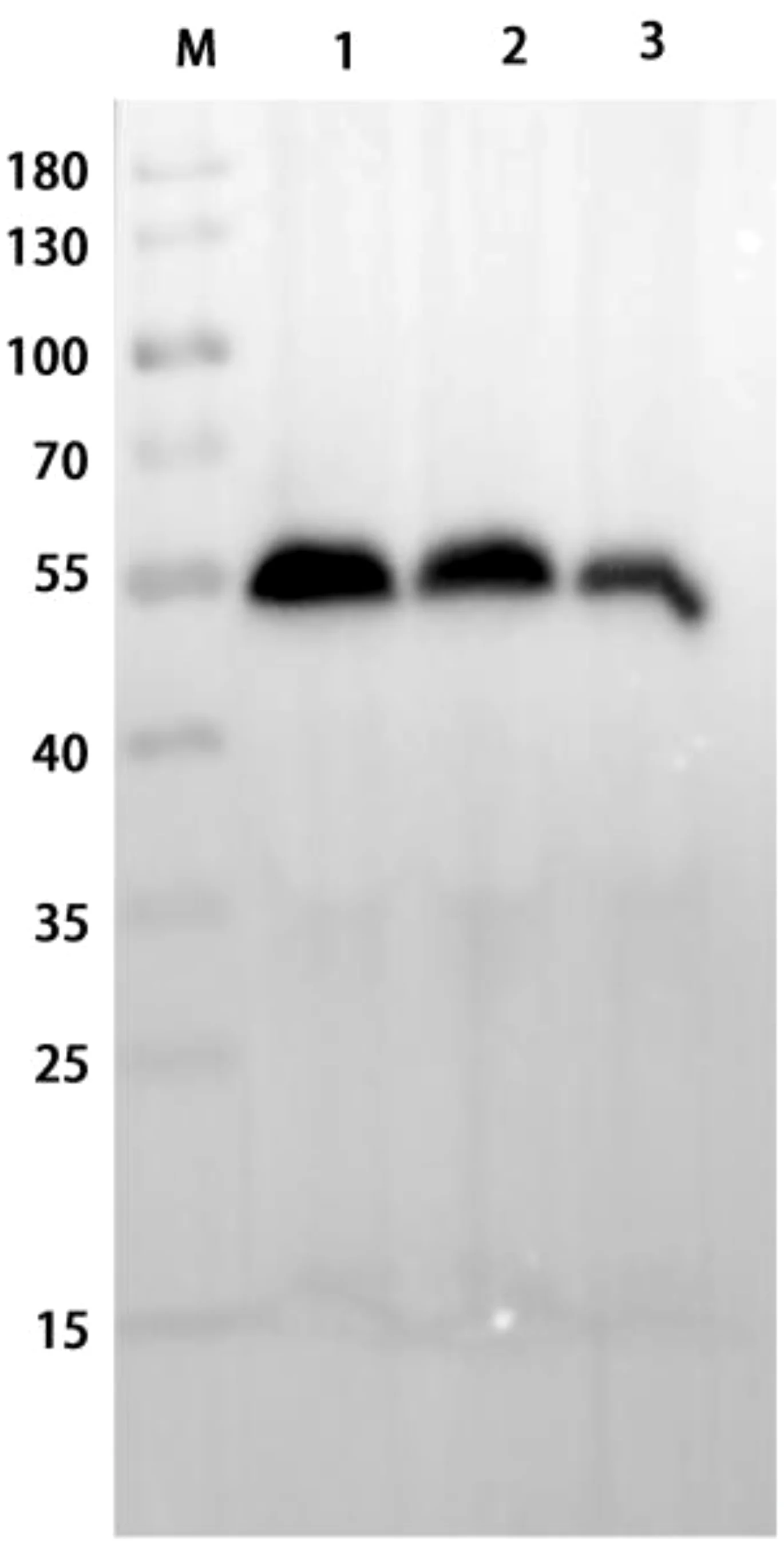
Purified protein was identified by His antibody. M: Protein molecular weight marker 1–3 CTLA4-IgV-EgG1Y162-2(4) protein.

#### 3.2.3 The immunoreactivity of CTLA4-IgV-EgG1Y162-2(4) protein was detected by Western blot

Western blot results showed that no target protein bands were found in normal mice and healthy individuals (**Figures 6A, C**). Target protein bands were found at approximately 53 kDa in infected mice and infected patients (**Figures 6B, D**). It was indirectly indicated that CTLA4-IgV-EgG1Y162-2(4)-specific antibodies could be produced in patients infected with *E. granulosus*, and the vaccine designed in this study had good immunogenicity and immunoreactivity.

**Figure 6:**
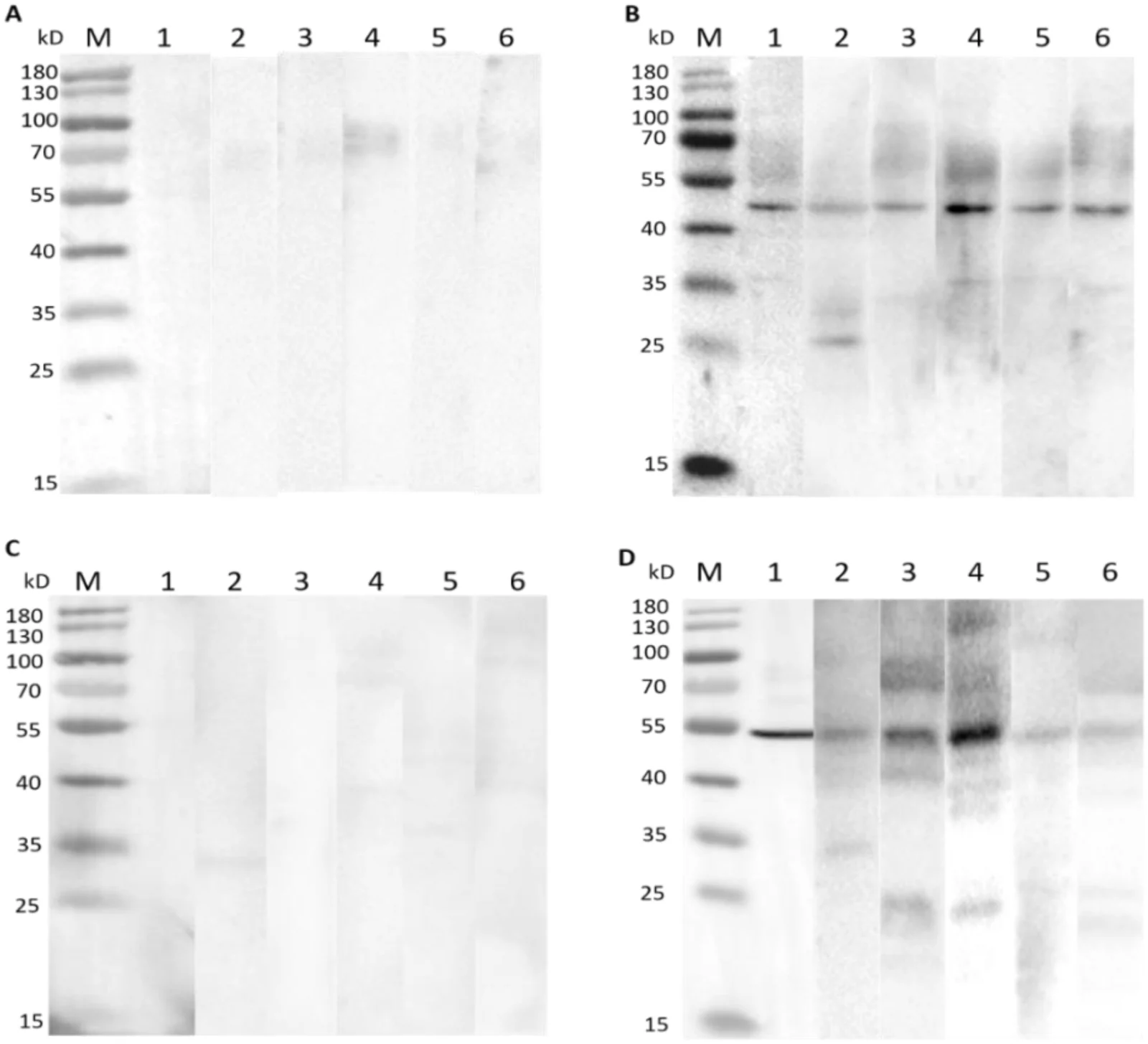
Results of antigenicity analysis of CTLA4-IgV-EgG1Y162-2(4) protein detected by Western blot. M: Protein molecular weight marker. **(A)** Lanes 1–6: Serum samples from normal mice. **(B)** Lanes 1–6: Serum samples from *Echinococcus*-infected mice. **(C)** Lanes 1–6: Serum samples from normal human. **(D)** Lanes 1–6: Serum samples from *Echinococcus*-infected human.

#### 3.2.4 Antigen capture ability and maturity of DCs

After 24 h co-culture of induced DCs with EgG1Y162-2(4) and CTLA4-IgV-EgG1Y162-2(4), flow cytometry results showed that the expression of MHC-II molecules and FITC-HIS in EgG1Y162-2(4) and CTLA4-IgV-EgG1Y162-2(4) groups were higher than those in normal groups. The percentage of MHC-II-positive DCs in the CTLA-4IgV-EgG1Y162-2(4) protein-stimulated group (17% ± 1.217%) was significantly higher than that in the EgG1Y162-2(4) protein-stimulated group (12.27% ± 0.379%) (*F* = 59.85, *p* < 0.01) (**Figure 2**-**4**). These results indicate that both proteins can promote DC differentiation, with CTLA-4IgV-EgG1Y162-2(4) protein showing a more pronounced effect, suggesting that it may possess stronger immunogenicity and thus enhance the antigen-presenting capacity of DCs (**Figure 7**).

**Figure 7:**
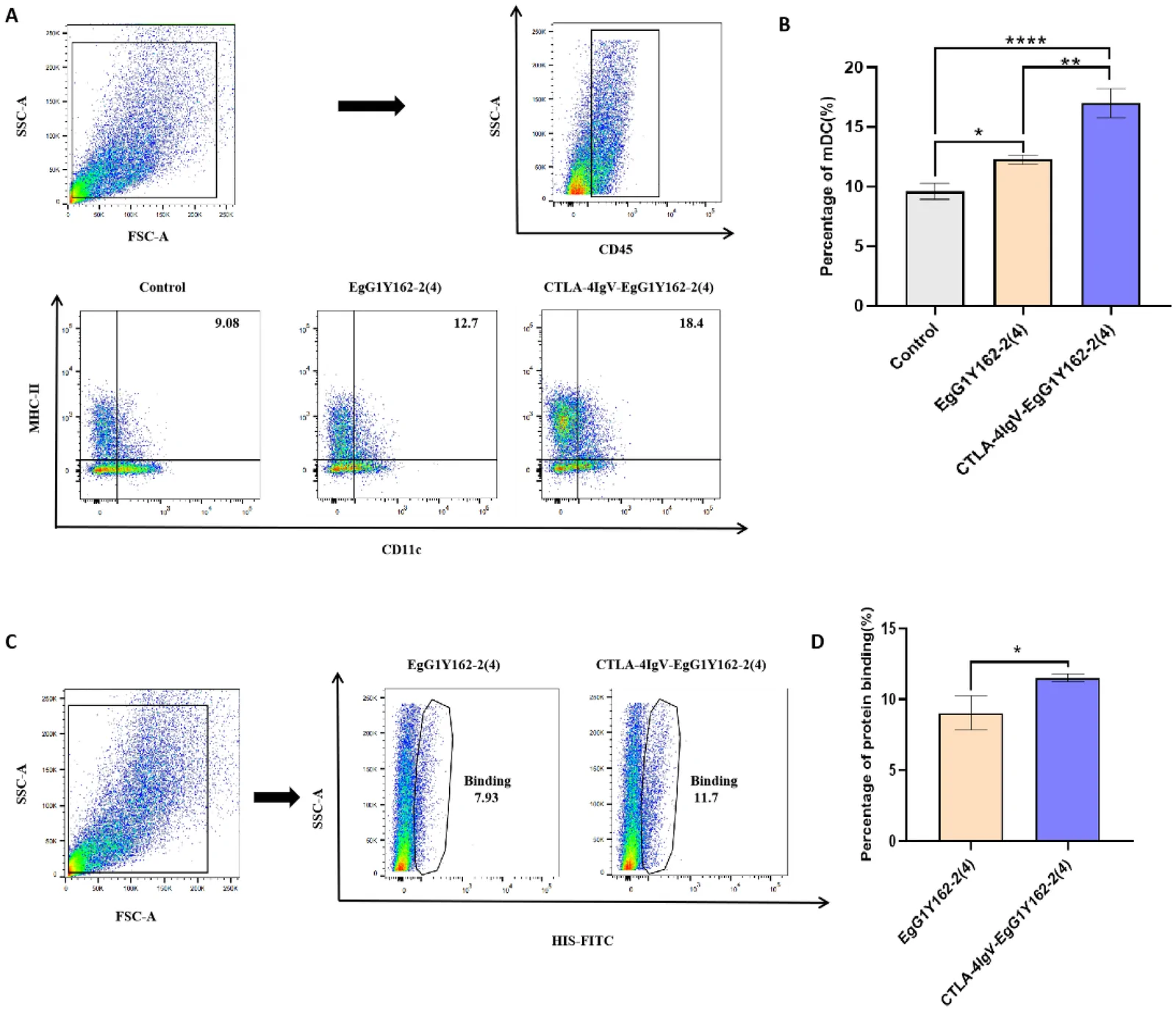
Co-culture of EgG1Y162-2(4) and CTLA-4IgV-EgG1Y162-2(4) proteins with DCs induced by bone marrow precursor cells from C57 mice. **(A)** Representative flow cytometry result plots of expression levels of MHC-II molecules. **(B)** Statistical analysis of MHC-II expression in each group. **(C)** Representative flow cytometry result plots of binding rates of EgG1Y162-2(4) and CTLA-4IgV-EgG1Y162-2(4) proteins to DCs **(D)** Statistical analysis of EgG1Y162-2(4) and CTLA-4IgV-EgG1Y162-2(4) protein binding to DCs. *P<0.05, **P<0.01, ****P<0.0001.

#### 3.2.5 DC maturity in immunized mice

To test whether CTLA-4IgV-EgG1Y162-2(4) protein can activate DCs in mice, we immunized four groups of mice. The DCs (CD11c^+^, CD86^+^, and MHC-II^+^) detected in the spleen of CTLA-4IgV-EgG1Y162-2(4) immunized mice were significantly higher than those in other groups. Compared with the blank control and negative control groups, the EgG1Y162-2(4) and CTLA-4IgV-EgG1Y162-2(4) protein-immunized groups showed higher expression rates of MHC-II and CD86 on CD11c^+^ cells (*p* < 0.05). The expression rates of MHC-II and CD86 in the EgG1Y162-2(4) group were 23.85% ± 0.887% and 19.50% ± 0.732%, respectively, while those in the CTLA-4IgV-EgG1Y162-2(4) group were 27.58% ± 1.378% and 22.33% ± 0.997%, respectively, both significantly higher than those in the EgG1Y162-2(4) group (*F* = 23.32, *p* < 0.05; *F* = 29.09, *p* < 0.05) (**Figure 8**). These results indicate that the addition of CTLA-4IgV increased the maturation rate of DCs, thereby enhancing their antigen-presenting capacity and inducing a stronger immune response.

**Figure 8:**
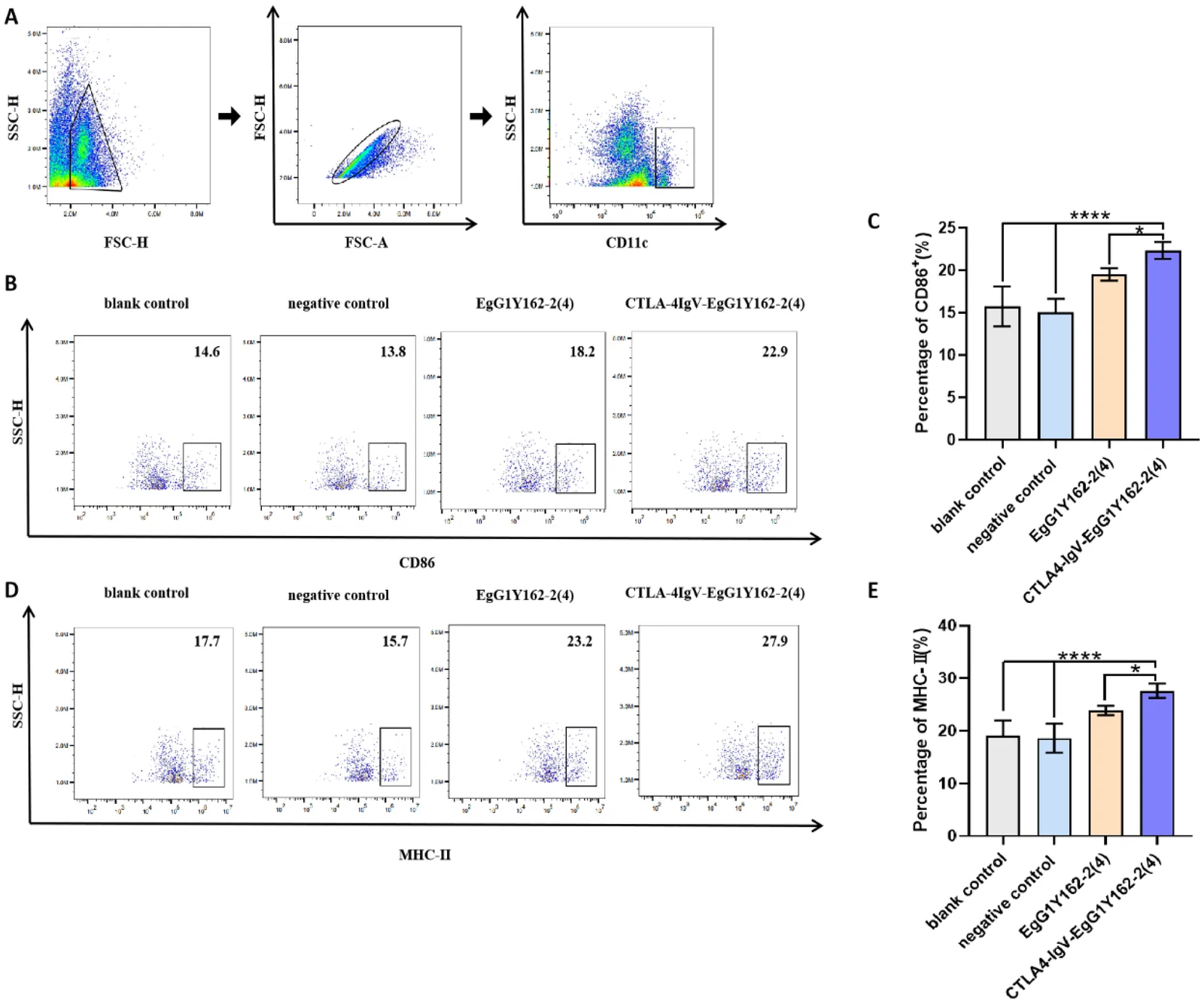
Maturation of splenic DCs induced by vaccine immunization of Balb/c mice. **(A)** Sorting loop gate strategy for DCs. **(B)** Representative flow cytometry plot for detection of CD86 expressed by DCs. **(C)** Statistical analysis of CD86 expression in each group. **(D)** Representative flow cytometry plot for the detection of MHC-II molecules expressed by DCs. **(E)** Statistical analysis of MHC-II expression in each group. *P<0.05, ****P<0.0001.

#### 3.2.6 Evaluation of spleen lymphocyte proliferation via the CCK-8 assay

CCK-8 detected the splenic proliferation capacity of mice, and the results showed that the splenic cell proliferation capacity of mice in the CTLA4-IgV-EgG1Y162-2(4) group was stronger than that in the EgG1Y162-2(4) group, and was stronger than that in both normal and negative control groups (**Figure 9A**). CTLA4-IgV-EgG1Y162-2(4)-immunized mice can promote the proliferation of lymphocytes and induce immune response.

**Figure 9:**
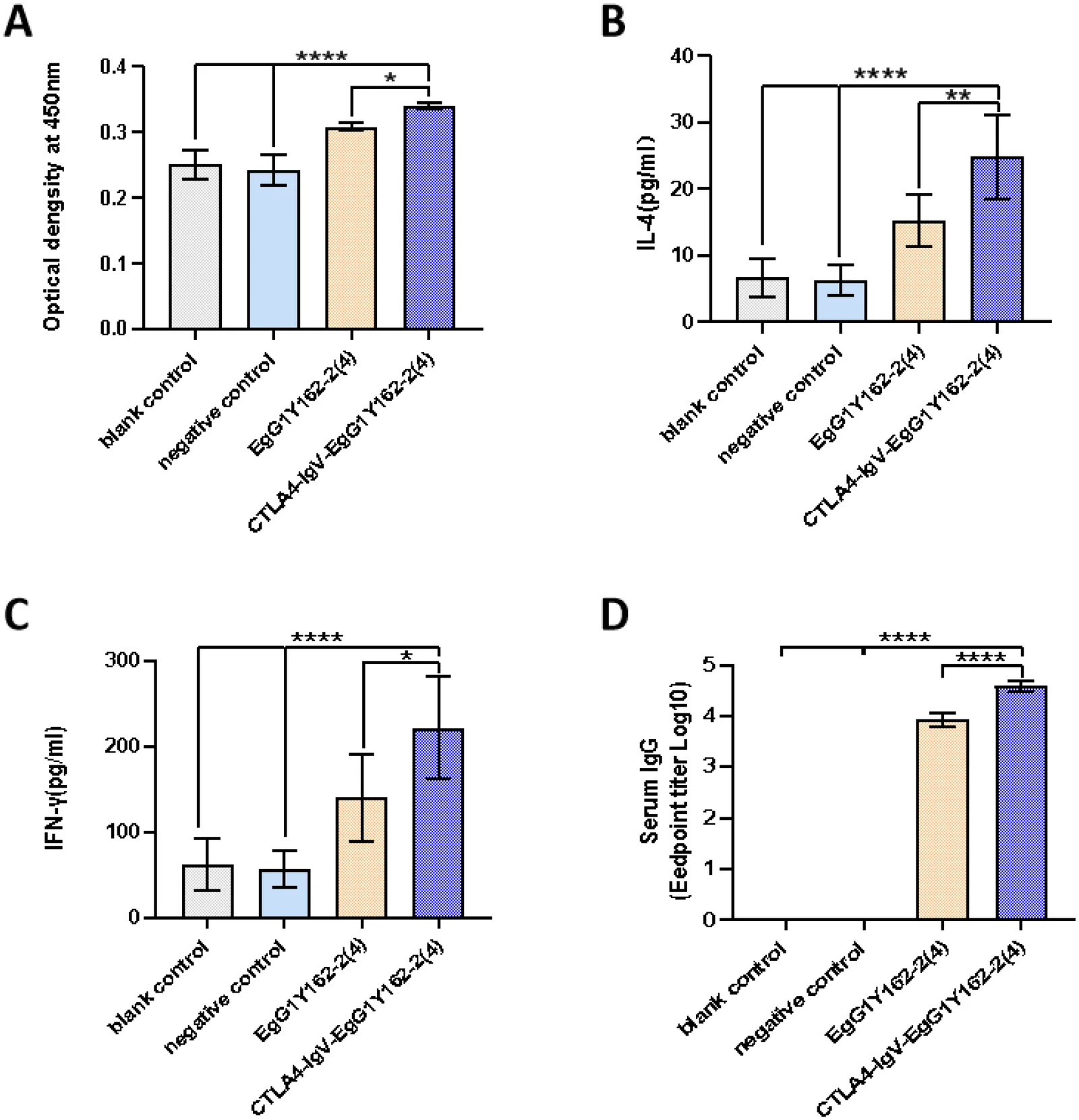
**(A)** The CCK-8 method was used to analyze the proliferative response of splenocytes after immunization of Balb/c mice. The bar graph shows the mean values of proliferative OD_450_ of splenocytes in all groups of immunized mice. **(B)** The concentration of IL-4 cytokines in the serum of each group of Balb/c mice was determined by ELISA. **(C)** The concentration of IFN-γ cytokines in the serum of each group of Balb/c mice was determined by ELISA. **(D)** The ELISA method was used to detect total IgG in BALB/c serum samples from all experimental groups. Serum samples were collected 6 weeks after immunization. The end-point titer was calculated as the reciprocal of the highest serum dilution having an OD at 450 nm 2.1 times higher than the critical value. *P<0.05, **P<0.01, ****P<0.0001.

#### 3.2.7 Quantification of IL-4 and IFN-γ via ELISA

To determine the ability of CTLA4-IgV-EgG1Y162-2(4) to induce cellular immune responses in mice, splenocytes were collected at week 6 of immunization stimulation, and the levels of IL-4 and IFN-γ cytokine secretion in the spleen of mice were measured by ELISA. The results showed that the secretion levels of IL-4 and IFN-γ in the CTLA4-IgV-EgG1Y162-2(4) group were higher than those in the EgG1Y162-2(4) group, and both were higher than those in the normal control and negative control groups (**Figures 9B,C**), which could induce a stronger cellular immune response in mice.

#### 3.2.8 Appraisal of specific protective antibody IgG following prophylactic intervention

ELISA detected the level of IgG antibody produced by each group of mice, and the result showed that mice in the vaccine immunization group produced neutralizing antibody IgG, EgG1Y162-2(4) recombinant antigen had a certain protective effect, and its potency was 1:1,350; CTLA4-IgV-EgG1Y162-2(4) recombinant antigen could induce mice to produce higher antibody titers (**Figure 9D**), and its potency was 1:4,050.

#### 3.2.9 Gross pathological changes of the liver and hydatid cysts in challenge-infected mice

At 120 days post-infection, the mice in each group were dissected, and gross pathological changes in the abdominal cavity and liver were observed and recorded (**Figure 10**). In the Eg model group, numerous free hydatid cysts were observed in the abdominal cavity, and the surface of the liver was covered with translucent milky-white cysts of varying sizes, ranging from 5 to 10 mm in diameter. In the control group, no cysts were observed in the abdominal cavity, and the liver surface was smooth and glossy, indicating successful establishment of the infection model in the challenge-infected mice. Compared with the Eg model group, both the number of free cysts in the abdominal cavity and the cyst burden on the liver surface were significantly reduced in each of the recombinant protein groups. Among these, the CTLA-4-IgV-EgG1Y162-2(4) group showed the most pronounced protective effect, with only a few small cysts (mostly less than 5 mm in diameter) on the liver surface, and the liver size and color were close to those of the normal control group.

**Figure 10:**
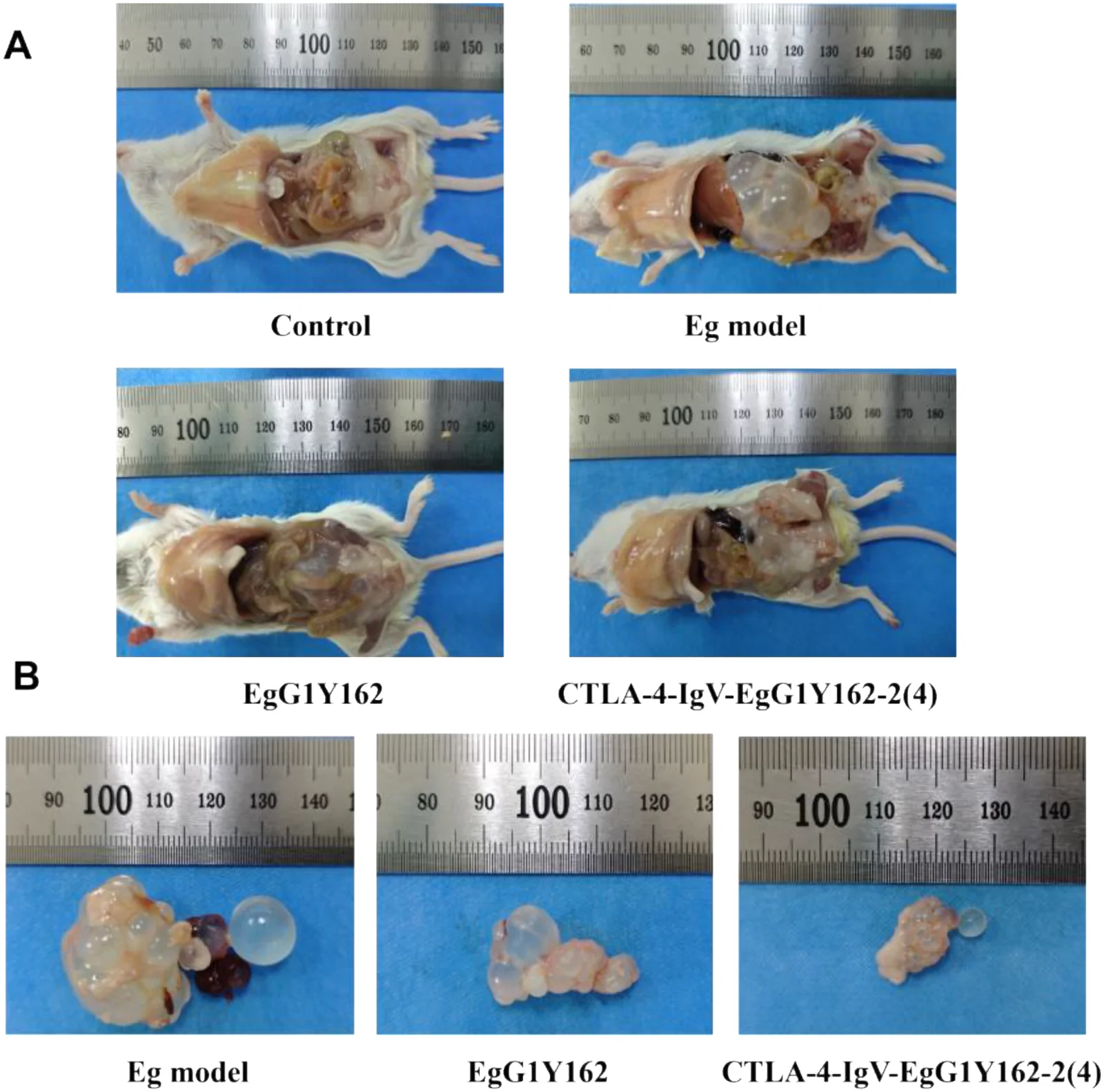
Pathological tissue changes in mice infected with parasites. **(A)** Gross photograph of *Echinococcus*-infected mice. **(B)** Photograph of hepatic lesion tissue from *Echinococcus*-infected mice.

#### 3.2.10 Infection rate and cyst wet weight inhibition rate in challenge-infected mice

To evaluate the protective efficacy of the vaccine against challenge infection with *E. granulosus*, the infection rate and the inhibition rate of hydatid cyst wet weight were statistically analyzed. All mice in the model group became infected, yielding an infection rate of 100%. Each of the vaccine groups exhibited a certain degree of protection, with the most pronounced effect observed in the CTLA-4-IgV-EgG1Y162-2(4) group, in which the infection rate decreased to 16.7%. Among the successfully infected mice, all vaccine groups significantly reduced the cyst wet weight (*p* < 0.001 vs. the model group). The inhibition rate of cyst wet weight in the CTLA-4-IgV-EgG1Y162-2(4) group reached 93.97% ± 14.78%, indicating that the introduction of CTLA-4-IgV further enhanced the vaccine’s ability to inhibit parasite development.

#### 3.2.11 Percentages of Th1 and Th2 cell subsets in splenocytes from challenge-infected mice

To clarify the type of immune response induced by vaccination at the cellular level, flow cytometry was used to determine the percentages of CD4^+^IL-4^+^ (Th2) and CD4^+^IFN-γ^+^ (Th1) cells in the spleens of challenge-infected mice (**Figures 11B, C**). The Eg model group showed a significantly increased Th2 percentage (3.34% ± 1.10%) and a decreased Th1 percentage (2.57% ± 0.54%), confirming Th2 polarization. All vaccine groups corrected this imbalance, with the CTLA-4-IgV-EgG1Y162-2(4) group showing the best outcome: the lowest Th2 percentage (2.04% ± 0.40%) and the highest Th1 percentage (4.13% ± 0.72%), significantly higher than those in the model group and the EgG1Y162 group (**Figures 11D, E**). These findings indicate that the CTLA-4-IgV-EgG1Y162-2(4) recombinant vaccine can effectively reverse the Th2-skewed immune response induced by Eg infection and elicit a stronger Th1-type cellular immune response.

**Figure 11:**
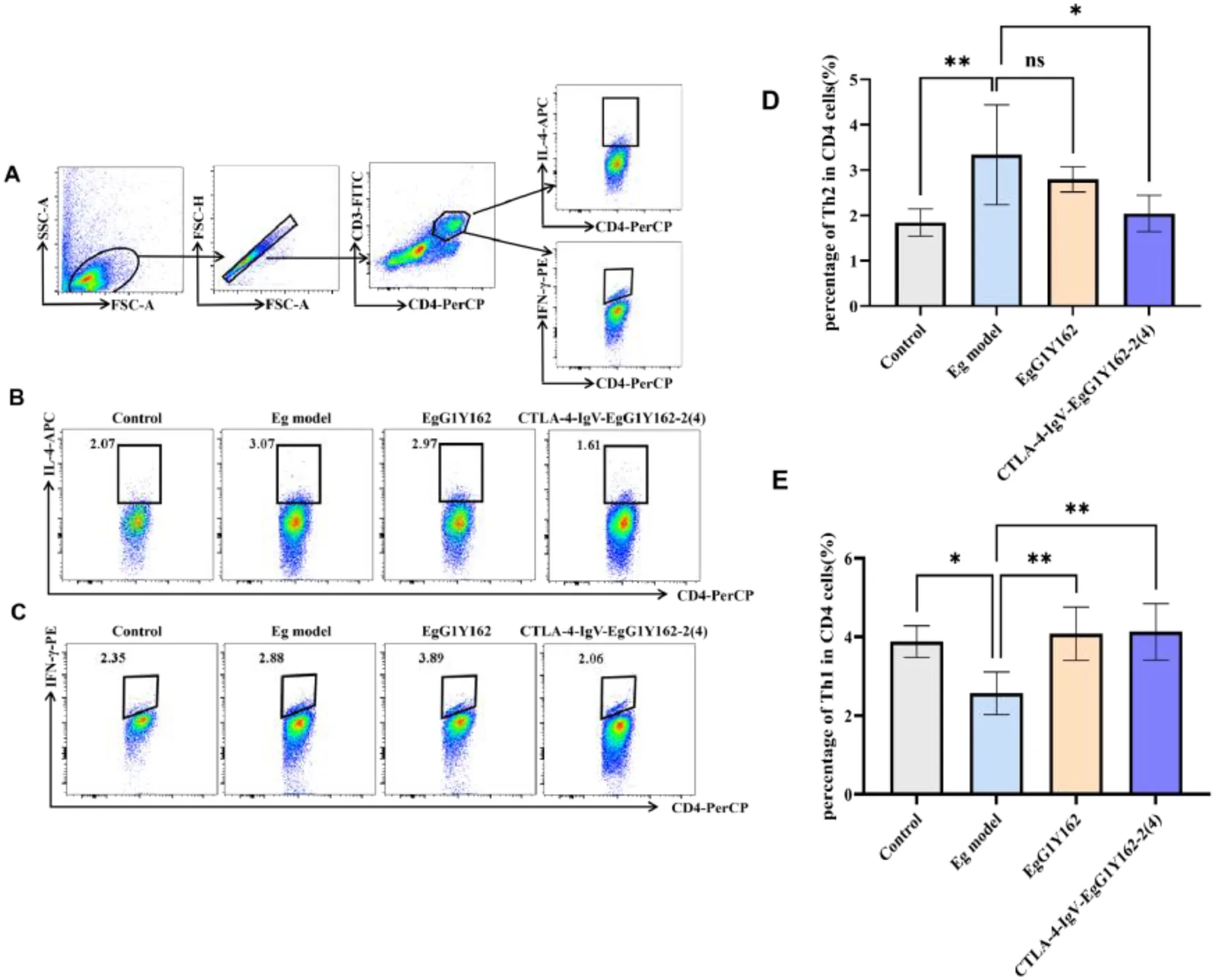
Percentage of CD4^+^IL-4^+^ T cells and CD4^+^IFN-γ^+^ T cells in the spleen of mice infected with parasites. **(A)** CD4^+^IL-4^+^ T-cell and CD4^+^IFN-γ^+^ T-cell gating strategy. **(B)** Flow cytometry representation of CD4^+^IL-4^+^ T cells. **(C)** Flow cytometry representation of CD4^+^IFN-γ^+^ T cells. **(D)** Statistical plot of CD4+IL-4^+^ T cells. **(E)** Statistical plot of CD4^+^IFN-γ^+^ T cells. *p < 0.05, **p < 0.01, ns *p* > 0.05.

#### 3.2.12 Serum secretion levels of IFN-γ and IL-4 in challenge-infected mice

To investigate the specific immune response following vaccination under challenge infection, serum levels of IFN-γ and IL-4 were measured. The Eg-infected model group exhibited a typical Th2-biased response, characterized by a significantly decreased IFN-γ level (3.16 ± 0.83 pg/mL) and a markedly elevated IL-4 level (28.78 ± 4.88 pg/mL). All vaccine groups reversed this bias, significantly reducing IL-4 while increasing IFN-γ. Among them, the CTLA-4-IgV-EgG1Y162-2(4) group showed the best outcome, with the highest IFN-γ level (9.74 ± 2.35 pg/mL) and the lowest IL-4 level (10.12 ± 4.26 pg/mL) (**Figure 12** and **Table 3**).

**Figure 12:**
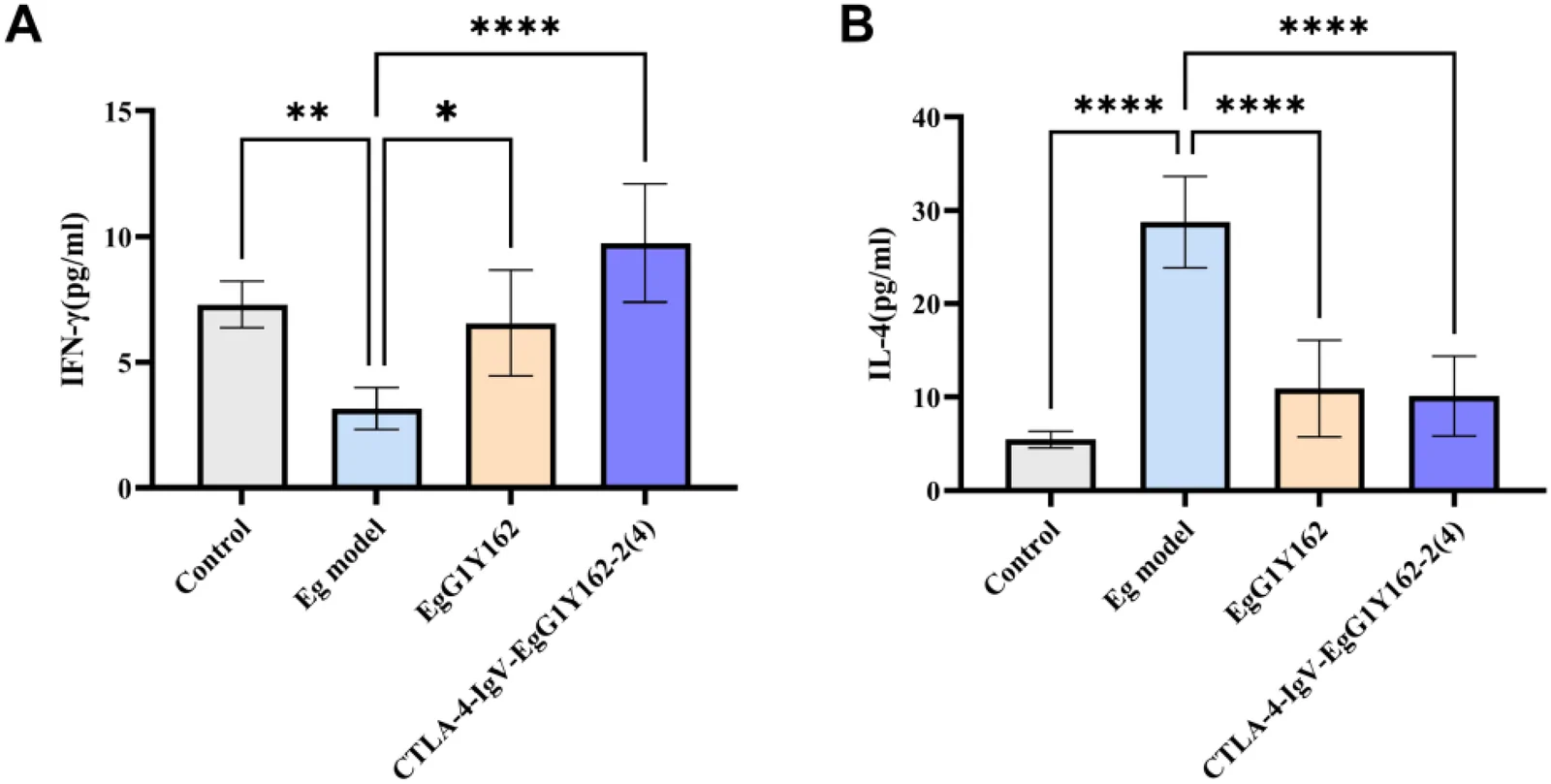
Serum IFN-γ and IL-4 secretion levels in mice infected with parasites. **(A)** ELISA assay for IFN-γ secretion levels in serum of mice from different groups. **(B)** ELISA assay for IL-4 secretion levels in serum of mice from different groups.*p < 0.05, ***p < 0.001, ****p < 0.0001.

**Table 3:** Statistics of cyst vesicle wet weight inhibition rate in parasite-infected mice.

Groups	Cyst wet weight inhibition rate (%)	*p-*value
EgG1Y162	72.57 ± 30.39^***^	*p* < 0.001
CTLA-4-IgV-EgG1Y162-2(4)	93.97 ± 14.78^***^	*p* < 0.001

Cyst wet weight inhibition rate = [1 − (cyst wet weight of experimental group/mean cyst wet weight of Eg model group)] × 100%. One-sample *t* test (one-tailed) vs. 0% (Eg model group). ***p < 0.001 vs. the Eg model group.

## 4 Discussion

CE is a typical zoonotic disease caused by human or animal infection with *E. granulosus*, which is the intermediate larval stage of *E. granulosus*. Its epidemic is becoming more and more widespread. Human infection with *E. granulosus* leads to the development of one or more hydatid cysts located most often in the liver and lungs, and less frequently in the bones, kidneys, spleen, muscles, and central nervous system. The severity of the disease varies depending on the number, location, and size of the cysts. Patients usually present with pain and impaired organ function, and the condition can worsen as the cyst expands. Some patients develop complications such as rupture of the capsule, infection, degeneration, and necrosis. In severe cases, it can cause secondary infection and anaphylactic shock, which can be life-threatening. At present, the treatment of CE at home and abroad is still based on surgery, supplemented by drug therapy. With the rapid development of surgery in recent times, the surgical methods for *E. granulosus* have been improved and innovated. Surgery aimed at increasing the eradication rate and reducing complications can be broadly divided into traditional, minimally invasive, and radical surgery. However, surgical treatment is still prone to recurrence, secondary diffuse CE, and complications (16). Mebendazole is the first benzimidazole found to have anti-CE activity *in vivo*, but associated with side effects such as anaphylaxis, hair loss, and reversible neutropenia (17). Albendazole is the drug of choice in the treatment of CE, and its bioavailability, although poor, is superior to other anti-infective drugs. Benzimidazole can cause hepatic and hematological toxicity and has been shown to be teratogenic in rats and rabbits (18), with many drug treatments causing more harm to patients than benefit. The pharmacokinetic properties, short half-life, and poor bioavailability of benzimidazoles hinder their inhibition against tissue-invasive and resident worms such as *E. granulosus*; all these factors highlight the urgent need to develop new and more effective compounds.

In recent years, the development of molecular biology technology has facilitated research on the prevention and control of parasitic diseases, and there has been significant progress on the research on recombinant parasite vaccines and DNA vaccines (19, 20), offering new strategies for the prevention and control of *E. granulosus*. The long-term nature and complexity of the control of *E. granulosus* dictate that simple, economical, and effective control measures should be used. By disrupting the pathogen’s cycle of transmission through immunoprophylaxis, the control process can be significantly shortened and the cost of control can be reduced (21). Vaccination to interrupt the transmission of the pathogen from host to host is an effective measure to cut off the epidemic chain of *E. granulosus* and is an ideal means to prevent human infection with *E. granulosus*. Vaccine design requires an accurate understanding of the molecular biology of *E. granulosus* and its antigens. The search for new therapeutic targets, screening, and identification of candidate genes are prerequisites and keys to the successful development of a vaccine against *E. granulosus*. The sequencing and publication of tapeworm DNA, stored in genomic databases, began in the late 1980s with the aim of cloning antigens for vaccine development and serological diagnosis (22, 23). Katoh et al. (24) identified the EmY162 antigen gene in their study of *E.m* cestodes and showed that EmY162 can target both mucosal and systemic immunity in dogs. Western blot analysis of sera from dogs with *E.m* showed that EmY162 exhibited a strong IgG antibody response, and that EmY162 has the potential to be used as a serological diagnostic antigen for *E.m* or vaccine candidates. Kouguchi et al. (25) found that the EmY162 recombinant antigen induced 74.2% immunoprotection in rats and that EmY162 was significantly reactive to serum from patients with AE, indicating that the EmY162 antigen is highly immunogenic in patients with AE. EmY162 antigen is considered an ideal vaccine candidate for *E.m*. Based on the question of whether similar antigens exist in *E. granulosus*, Cao et al. (10) cloned the EgG1Y162 antigen gene from *E. granulosus* and showed that EgG1Y162 has high similarity to EmY162 in gene sequence, amino acid sequence, and protein structure, suggesting its promise as a protective vaccine candidate for *E. granulosus*.

The development of anti-*E. granulosus* vaccine is challenging. This is primarily due to the parasite’s long-term parasitism and evolution within the host, which have led to sophisticated immune escape mechanisms that complicate bioinformatics-driven vaccine development. In addition, *E. granulosus* is a multicellular organism, and a single subunit protein vaccine is difficult to induce a high level of immune protection in the host. In view of the advantages of the recombinant multi-epitope vaccine, the biological characteristics of EgG1Y162 antigen gene, and the characteristics of the CTLA-4 extracellular region targeting DCs, we intend to design a recombinant multi-epitope vaccine CTLA-4IgV-EgG1Y162-2(4) targeting DCs. DCs are the major APCs as a result of their ability to cross-represent extracellular antigen via MHC class I molecule and potency to promote T-cell proliferation. Studies have shown that targeting DCs can improve antigen uptake and immune response (26, 27). CTLA-4 is a T-cell transmembrane receptor that includes the extracellular V region, transmembrane region, and intracytoplasmic tail (28); CTLA4-IgV is its extracellular region, and CTLA4-IgV is present in the B7 molecular binding sequence MYPPPY, which has the ability to bind to APCs. Using CTLA4-IgV as a targeting ligand for APCs, CTLA4-IgV fused to the specific antigen EgG1Y162-2(4) can target specific antigen binding to APCs as a way to enhance the immune response of epitope vaccines against CE. It has been shown that specific antigen targeting by CTLA-4 extracellular structural domain fusion antigen can be presented to APCs to enhance the adaptive immune response of the organism (29). Since the immunogenicity of a single epitope is weak, T-cell–B-cell combination epitope can induce both a strong humoral and cellular immune response (30). We selected the EgG1Y162–2 antigen gene rich in T-cell and B-cell epitopes and connected four EgG1Y162–2 fragments through the “GSGGSG” linker sequence to increase their epitopes, so that the tandem EgG1Y162–2 could be folded normally, and the recombinant multi-epitope vaccine EgG1Y162-2(4) was prepared. Using the pET30a-CTLA-4IgV-EgG1Y162 and pET30a-EgG1Y162-2(4) preserved in our laboratory, the recombinant plasmid of pET30a-CTLA-4IgV-EgG1Y162-2(4) has been successfully constructed and the protein was expressed in this study. The tertiary structure model of the recombinant protein CTLA-4IgV-EgG1Y162-2(4) was obtained by SWISS-MODEL online software analysis, which showed that the CTLA-4IgV protein and EgG1Y162-2(4) protein could overcome the effect of spatial block and fold correctly, thus allowing the recombinant protein to perform its immune function normally. This study utilized IEDB and SYFPEITHI for predicting the antigenic epitopes of the CTLA-4IgV-EgG1Y162-2(4) recombinant antigen. The results revealed that the recombinant antigen possesses six co-associated T-cell and B-cell epitopes, with no epitope displacement observed. Furthermore, the augmentation of these T-cell and B-cell co-associated epitopes enhances the immunogenicity of the CTLA-4IgV-EgG1Y162-2(4) recombinant multi-epitope vaccine. Bioinformatics is currently more applied in protein structure and function, which can accurately analyze the antigenic epitopes of proteins and improve efficiency, and is widely used in vaccine development. With the development of immunoinformatics at home and abroad, immunoinformatics data are being improved and provide a good platform for the design of recombinant multi-epitope vaccines.

Multi-epitope vaccines are a new type of vaccine developed in recent decades, and their immune response rate and safety have been shown to be higher than traditional vaccines in early clinical trials (31). Multi-epitope vaccines can enhance specificity and immune response based on conserved epitopes in antigen proteins and avoid adverse reactions caused by unnecessary epitopes (32). The immune efficacy of recombinant multi-epitope vaccines needs to be validated by a series of immunological test methods. Appel et al. (33) and Schuijs et al. (34) showed that DCs are immune cells with the strongest antigen presenting ability. Mature DCs can effectively activate naive T cells and are crucial for initiating adaptive immune response, which can be obtained from peripheral blood mononuclear cells, bone marrow, and spleen. After the immune system is activated, Th0 differentiates into Th1 and Th2 under the stimulation of cytokines such as IL-12 and IL-4, which then mediates cellular immune response and MHC-II-restricted CD4^+^ Th2 response, inducing the body to produce immune response (35). In this study, bone marrow cell suspension derived from C57 mice was extracted and induced to differentiate into DCs using rmGM-CSF and rmIL-4 cytokine. The immunophenotype of DCs was detected by flow cytometry, and it was found that the maturity and binding rate of DCs in the CTLA-4IgV-EgG1Y162-2(4) group was higher than that in the EgG1Y162-2(4) group. The results indicated that CTLA-4IgV-EgG1Y162-2(4) protein could effectively stimulate the maturation of DCs. As the settlement site of immune cells, the spleen is an important part of the body’s immune response to the stimulation of foreign substances such as antigens. When antigenic components are sensed by the body’s immune system, the proliferation of splenic lymphocytes can be initiated at an early stage, and these proliferating lymphocytes will secrete a variety of cytokines and antibodies to neutralize the foreign substances and regulate the immune response. Therefore, the proliferation of splenic lymphocytes can indirectly reflect the immune status of the body. In this study, the proliferation level of splenic lymphocytes and the secretion level of IFN-γ, IL-4, and IgG were measured in BALB/c mice immunized with the vaccine for 6 weeks, and the results showed that the proliferation level of splenic lymphocytes and the secretion level of cytokines and antibodies in the group immunized with the CTLA-4IgV-EgG1Y162-2(4) protein were higher than those in the other groups, which could have an immune-protecting effect on the organism. The BALB/c mouse immunization assay further confirmed that the CTLA-4IgV-EgG1Y162-2(4) recombinant protein induced specific immunoprotective effects in mice and had positive biological activity.

Challenge experiments are a critical means to evaluate the protective efficacy of vaccines against *E. granulosus* and to elucidate the immune mechanisms, serving as a “touchstone” for clinical translation of vaccines. In this study, mice immunized with recombinant protein vaccines were challenged by intraperitoneal injection of protoscoleces. At 120 days post-infection, partial mice in the model group were euthanized for observation, and the infection rate reached 100%, indicating successful establishment of the Eg infection model. Compared with the model group, all immunized groups showed varying degrees of reduction in infection rate and lesion wet weight, with the most pronounced reduction observed in the CTLA-4-IgV-EgG1Y162-2(4) group. Because of limited cage space resulting in a small number of mice per group, the infection rate statistics had some random error; however, combined assessment of cyst wet weight inhibition rate and infection rate indicated that the recombinant protein vaccine conferred protective efficacy in mice. Flow cytometric analysis of Th1 and Th2 subsets in the spleen revealed that the percentage of Th1 cells in the Eg model group showed a decreasing trend compared with other groups and was significantly lower than that in the CTLA-4-IgV-EgG1Y162-2(4) group. Meanwhile, the percentage of Th2 cells in the model group was significantly higher than that in other groups. These results indicate that chronic *E. granulosus* infection disrupts the Th1/Th2 immune balance in mice, shifting towards a Th2 bias, which is consistent with previous reports: chronic parasitic infection in the late stage induces a non-protective Th2-type immunity that facilitates immune escape. Recombinant protein vaccines can effectively correct this immune imbalance, promote the restoration of protective immune responses, inhibit parasite growth, and reduce both the infection rate and hydatid cyst wet weight.

There are certain limitations in this study. First, the spatial structure of the recombinant protein in this study was predicted by homology modeling (Swiss Model), without empirical structural validation such as x-ray crystallography or cryo-electron microscopy. Although such bioinformatics tools are highly reliable, the actual physical folding of the fusion protein *in vitro* or *in vivo* may differ from the predicted structure, which could affect epitope presentation and the subsequent immune response. Therefore, the conclusions drawn from computer-simulated structural predictions in this study still require validation through experimental structural determination. IgG subclass analysis could not be performed because the volume of serum collected from each mouse was insufficient for both cytokine detection and subclass typing, thereby limiting a more detailed assessment of Th1/Th2 polarization. Second, precise quantification of residual endotoxin in the purified recombinant protein was not carried out, although a wash buffer containing 60% isopropanol was used to remove endotoxins during purification. Third, ideally, an adjuvant-only control group should have been included to exclude the potential interference of non-specific immune responses induced by the adjuvant itself. However, owing to the limited availability of animal housing space in our facility and the prolonged duration of the *E. granulosus* infection model adopted in this study, we were unable to complete this control group within the current experimental timeline. Furthermore, our pilot experiments revealed that subcutaneous injection of complete Freund’s adjuvant (CFA) alone caused severe local skin ulceration and persistent pain in mice, necessitating early termination of these animals for ethical and welfare considerations; therefore, this group was not incorporated into our final experimental design. Despite this limitation, we have taken alternative measures to minimize its impact on data interpretation, as detailed in the Results section. We will include a dedicated adjuvant-only control in our future studies to more comprehensively evaluate the immunomodulatory effects of the vaccine formulation. Nevertheless, the absence of this control does not compromise our major conclusions. In ELISA, an irrelevant tag protein served as a negative control, and post-immune sera showed specific reactivity only to the vaccine antigen, with OD values against the irrelevant protein comparable to naïve sera (*p* > 0.05), confirming antigen-specific antibody responses. In flow cytometry, unstimulated wells were used as background controls, and antigen-specific IFN-γ and IL-4 secretion in the vaccinated group was significantly higher than background (*p* < 0.05), far exceeding reported adjuvant-only background values (**Table 4**). Collectively, these controls have sufficiently ruled out non-specific immune interference. A dedicated adjuvant-only control will be included in our future studies to more comprehensively evaluate the vaccine’s immunomodulatory effects. Fourth, the sample size of the *in vivo* challenge experiment was relatively limited (*n* = 6 per group; see Section 2.2.6.1 and **Table 5**). Deriving a precise inhibition rate of cyst wet weight (e.g., 93.97% ± 14.78%) from such a small sample population is statistically unreliable, and the limited sample size may introduce random error into the statistical results of efficacy. Therefore, the above efficacy data should be interpreted with caution and require further validation in larger sample populations.

**Table 4:** Statistical analysis of serum IFN-γ and IL-4 levels in mice infected with the parasite ( $\bar{\text{x}}$ ± s, *n* = 6).

Groups	IFN-γ (pg/mL)	IL-4 (pg/mL)
Control	7.30 ± 0.92^*^	5.47 ± 0.87^****^
Eg model	3.16 ± 0.83	28.78 ± 4.88
EgG1Y162	6.60 ± 2.10	10.93 ± 5.17^****^
CTLA-4-IgV-EgG1Y162-2(4)	9.74 ± 2.35^***^	10.12 ± 4.26^****^
*F*	38.40	34.00

*P<0.05, ***P<0.001, ****P<0.0001 vs the Eg model group.

**Table 5:** Statistics of infection rate in mice infected with Eg.

Groups	Infected	Uninfected	Infection rate (%)	*p-*value
Eg model	6	0	100	
EgG1Y162	4	2	66.7	vs. Eg model: 0.455
CTLA-4-IgV-EgG1Y162-2(4)	1	5	16.7	vs. Eg model: 0.015

Infection status was determined based on the presence or absence of lesions in the peritoneal cavity of the mice. Overall comparison was performed using Fisher’s exact test; pairwise comparisons were performed using Fisher’s exact test with Bonferroni correction (α’ = 0.0167).

CFA and incomplete Freund’s adjuvant (IFA) were employed as immunological adjuvants in this study. Both CFA and IFA are classic and potent adjuvants capable of effectively enhancing humoral and cellular immune responses, and are commonly used in preclinical studies as positive controls or as immune-stimulating tools for proof-of-principle investigations. CFA contains inactivated *Mycobacterium tuberculosis* and induces a strong Th1-type immune response, whereas IFA lacks the mycobacterial component and exerts a relatively milder immune-stimulating effect, and is typically used for booster immunizations. However, both adjuvants are known to cause adverse reactions to varying degrees. Studies have shown that CFA can induce local skin ulceration and persistent pain in mice, which is closely associated with the strong local inflammatory response induced by CFA. Although IFA is less toxic than CFA, it may still cause injection-site inflammation and granuloma formation. Given that safety and tolerability are primary considerations in the clinical translation of vaccines, the toxic reactions induced by CFA and IFA suggest that such adjuvants are difficult to directly apply in humans. Although both adjuvants can effectively enhance immune responses in experimental animals, their local inflammatory reactions and risk of tissue damage limit their clinical applicability. Therefore, future studies should focus on identifying safer and better-tolerated alternative delivery systems or adjuvant strategies, such as the Montanide ISA series, CpG oligonucleotides, poly(lactic-co-glycolic acid) (PLGA) nanoparticles, or liposomal delivery systems, to reduce local and systemic toxicity while maintaining immune efficacy. The feasibility of these alternatives still requires validation through systematic safety evaluation and clinical trials.

In conclusion, this study demonstrated that the recombinant CTLA-4IgV-EgG1Y162-2(4) vaccine candidate elicited protective efficacy in a murine model of *E. granulosus* infection. However, several important directions remain for future investigation. First, given that the natural intermediate hosts (e.g., sheep) and definitive hosts (e.g., dogs) differ substantially from the murine model, future studies must incorporate *in vivo* testing of this vaccine candidate in these natural hosts to confirm its protective efficacy and translational potential. Second, future research should focus on evaluating the long-term memory immune response, as assessing specific antibody titers and cellular immunity over a period significantly longer than the current 120-day challenge timeline is required to determine the durability of the vaccine’s protection. Third, to advance this vaccine candidate toward practical and clinical application, future work should include formulation with safer, commercially viable adjuvants to replace CFA and IFA, and to evaluate whether strong immunogenicity is maintained without the associated severe adverse effects. Fourth, as the natural route of *E. granulosus* infection is oral/gastrointestinal, future studies should explore mucosal immunization routes (e.g., oral or intranasal delivery), and evaluating the induction of secretory IgA in the gut region would be highly valuable, as mucosal antibodies may neutralize parasite oncospheres before they penetrate the host’s intestinal barrier. Finally, future translational work should include empirical *in vitro* stability testing (e.g., thermal shift assays and degradation over time) to support the bioinformatics predictions of the instability index and theoretical physicochemical properties of the vaccine, as demonstrating a long shelf life is critical for a vaccine intended for deployment in remote, endemic agricultural regions where cold-chain logistics might be challenging.

## Data Availability

The original contributions presented in the study are included in the article/supplementary material. Further inquiries can be directed to the corresponding authors.
